# BACE1 elevation engendered by GGA3 deletion increases β-amyloid pathology in association with APP elevation and decreased CHL1 processing in 5XFAD mice

**DOI:** 10.1186/s13024-018-0239-7

**Published:** 2018-02-02

**Authors:** WonHee Kim, Liang Ma, Selene Lomoio, Rachel Willen, Sylvia Lombardo, Jinghui Dong, Philip G. Haydon, Giuseppina Tesco

**Affiliations:** 1 Alzheimer’s Disease Research Laboratory, Tufts University School of Medicine, 136 Harrison Avenue, Boston, MA 02111 USA; 2 Department of Neuroscience, Tufts University School of Medicine, 136 Harrison Avenue, Boston, MA 02111 USA

**Keywords:** Alzheimer disease, Amyloid-beta (Aβ), Beta-secretase 1 (BACE1), Golgi-localized γ-ear-containing ARF binding protein 3 (GGA3), Amyloid precursor protein (APP), Cell adhesion molecule L1 like protein (CHL1), Down syndrome

## Abstract

**Background:**

β-site amyloid precursor protein cleaving enzyme 1 (BACE1) is the rate-limiting enzyme in the production of amyloid beta (Aβ), the toxic peptide that accumulates in the brains of Alzheimer’s disease (AD) patients. Our previous studies have shown that the clathrin adaptor Golgi-localized γ-ear-containing ARF binding protein 3 (GGA3) plays a key role in the trafficking of BACE1 to lysosomes, where it is normally degraded. GGA3 depletion results in BACE1 stabilization both in vitro and in vivo. Moreover, levels of GGA3 are reduced and inversely related to BACE1 levels in post-mortem brains of AD patients.

**Method:**

In order to assess the effect of GGA3 deletion on AD-like phenotypes, we crossed GGA3 −/− mice with 5XFAD mice. BACE1-mediated processing of APP and the cell adhesion molecule L1 like protein (CHL1) was measured as well as levels of Aβ42 and amyloid burden.

**Results:**

In 5XFAD mice, we found that hippocampal and cortical levels of GGA3 decreased while BACE1 levels increased with age, similar to what is observed in human AD brains. GGA3 deletion prevented age-dependent elevation of BACE1 in GGA3KO;5XFAD mice. We also found that GGA3 deletion resulted in increased hippocampal levels of Aβ42 and amyloid burden in 5XFAD mice at 12 months of age. While levels of BACE1 did not change with age and gender in GGAKO;5XFAD mice, amyloid precursor protein (APP) levels increased with age and were higher in female mice. Moreover, elevation of APP was associated with a decreased BACE1-mediated processing of CHL1 not only in 12 months old 5XFAD mice but also in human brains from subjects affected by Down syndrome, most likely due to substrate competition.

**Conclusion:**

This study demonstrates that GGA3 depletion is a leading candidate mechanism underlying elevation of BACE1 in AD. Furthermore, our findings suggest that BACE1 inhibition could exacerbate mechanism-based side effects in conditions associated with APP elevation (e.g. Down syndrome) owing to impairment of BACE1-mediated processing of CHL1. Therefore, therapeutic approaches aimed to restore GGA3 function and to prevent the down stream effects of its depletion (e.g. BACE1 elevation) represent an attractive alternative to BACE inhibition for the prevention/treatment of AD.

**Electronic supplementary material:**

The online version of this article (10.1186/s13024-018-0239-7) contains supplementary material, which is available to authorized users.

## Background

Alzheimer’s disease (AD) is a progressive neurodegenerative disorder characterized by memory impairments and cognitive deterioration. AD is characterized by the cerebral accumulation of amyloid beta (Aβ), a ~ 4 kDa peptide formed by the serial proteolysis of amyloid precursor protein (APP), by the β- and γ-secretases. β-site amyloid precursor protein cleaving enzyme 1 (BACE1), a membrane-tethered aspartyl protease, has been identified as the β-secretase responsible for APP cleavage [1–4]. BACE1 is an N-glycosylated type 1 transmembrane protein that undergoes constitutive N-terminal processing in the Golgi apparatus. The ectodomain contains four glycosylation sites and two signature sequences typically associated with aspartyl proteases (DT/SGT/S) [5]. BACE1 is targeted through the secretory pathway to the plasma membrane where it is internalized to the endosomes. BACE1 is then trafficked back to the cell surface or trans-Golgi network (TGN) through recycling endosomes, or transported to the lysosomes for degradation [6]. Our previous studies have shown that BACE1 is degraded via the lysosomal pathway [7], and that depletion of the clathrin adaptor Golgi-localized γ-ear-containing ARF binding protein 3 (GGA3) results in increased BACE1 levels and activity due to its impaired lysosomal trafficking and degradation [8, 9]. We further demonstrated the regulatory role of GGA3 on BACE1 in vivo by showing that BACE1 levels are increased in the brain of GGA3−/− mice [10]. We also determined that depletion of GGA3 naturally occurs following caspase activation both in cellular models of apoptosis and in rodent models of stroke and traumatic brain injury (TBI) [8, 10]. More importantly, we discovered that levels of GGA3 are decreased and inversely correlated with BACE1 levels in post-mortem AD brains [8].

In order to assess the effect of GGA3 deletion on AD-like phenotypes, we crossed GGA3 −/− mice with 5XFAD mice to generate six different genotypes GGA3+/+;5XFAD- (GGA3WT), GGA3+/−;5XFAD- (GGA3Het), GGA3−/−;5XFAD- (GGA3KO) GGA3+/+;5XFAD+ (GGA3WT;5XFAD), GGA3+/−;5XFAD+ (GGA3Het;5XFAD), and GGA3−/−;5XFAD+ (GGA3KO;5XFAD). The 5XFAD mice overexpress mutant human APP(695) with the Swedish (K670 N, M671 L), Florida (I716V), and London (V717I) Familial Alzheimer’s Disease (FAD) mutations along with human PS1 harboring two FAD mutations, M146 L and L286 V. We found that levels of GGA3 were decreased while BACE1 levels increased with age in 5XFAD mice, similar to what is observed in human AD brains. Moreover, the deletion of GGA3 prevented such time-dependent increase of BACE1 in 5XFAD mice, suggesting that GGA3 plays a key role in the elevation of BACE1 observed in 5XFAD mice and human AD brains. While levels of BACE1 did not change with age in GGA3KO;5XFAD mice, levels of Aβ42 and amyloid burden were increased in the hippocampus of GGA3KO;5XFAD mice compared to GGA3WT;5XFAD littermates at 12 months of age, particularly in female mice. The increase in amyloid burden was associated with increased levels of APP and decreased BACE1-mediated processing of CHL1 in GGA3KO;5XFAD compared to GGA3WT;5XFAD mice, particularly in female mice.. Such an effect occurs likely because transgenic APP, which accumulated with an age- and gender-dependent manner in 5XFAD mice, outcompetes CHL1 for BACE1-mediated cleavage. In order to further support these findings, we analyzed hippocampal tissue from subjects affected by Down Syndrome (DS) carrying an extra copy of chromosome 21. The APP gene lies on chromosome 21 and as a consequence, levels of APP are increased in DS patients compared to controls. We found that BACE1-mediated processing of CHL1 was decreased in brains from DS patients compared to controls. Altogether these results indicate that increased APP levels reduce BACE1-mediated processing of CHL1 processing in both 5XFAD and human DS brains, most likely owing to substrate competition.

## Methods

### Mice handling and breeding

GGAKO mice have been previously described [10]. The 5XFAD mice co-overexpress familial AD mutant forms of human APP695 with Swedish (K670 N, M671 L), Florida (I716V), and London (V717I) mutation, and presenilin 1 (PS1) with M146 L and L286 V mutation under transcriptional control of the neuronal-specific mouse Thy-1.2 promoter [11]. The 5XFAD mouse line Tg6799 was purchased from the Jackson Laboratories (MMRRC Stock No: 34840-JAX). This line is on the B6/SJL genetic background and develops intraneuronal Aβ42 accumulation starting at 1.5 months of age, just prior to amyloid deposition and gliosis, which begins at 2 months of age. On a congenic C57BL/6 J genetic background (MMRRC stock 34848) it has been the observation of the MMRRC that this phenotype is not as robust as that demonstrated in the B6SJL hybrid background. Hemizygous 5XFAD transgenic mice, on a genetic background of B6/SJL, were crossed to GGA3KO mice, on a genetic background of C57BL/6, to yield GGA3Het;5XFAD male and GGA3Het female mice. Both mice were intercrossed to generate six different genotypes: GGA3WT, GGA3Het, GGA3KO, GGA3WT;5XFAD, GGA3Het;5XFAD, and GGA3KO;5XFAD. The resulting offspring were genotyped by PCR analysis of genomic DNA from tail biopsies. BACE1−/− (BACE1KO) mice were purchased from the Jackson Laboratories (Stock No: 004714). All animal experiments were performed with the approval of Tufts University Institutional Animal Care and Use Committees.

### Immunoblot analysis

Mice were anesthetized with isoflurane and transcardially perfused with PBS buffer. One collected hemibrain was used for biochemical analysis and the other for histological analysis. Hippocampus and cortex were rapidly dissected from the hemibrain for biochemical analysis and snap frozen in liquid nitrogen. The snap frozen samples were homogenized in 4 volumes of PBS buffer containing 1X Protease inhibitor cocktail and 1X Phosphatase inhibitor cocktail (Thermo Scientific). Brain homogenates in PBS were separated for immunoblot analysis and ELISA. For immunoblot analysis, 2 times concentrated RIPA (radioimmunoprecipitation assay) buffer (150 mM NaCl, 1% NP-40, 0.5% sodium deoxycholate, 0.1% SDS, 1 mM EDTA, and 50 mM Tris HCl, pH 7.4) containing protease and phosphatase inhibitors were added to brain homogenates in PBS, sonicated briefly, and then incubated for 30 min on ice. The homogenates were centrifuged at 14,000×g for 20 min at 4 °C and then the supernatants were collected for immunoblot analysis. Protein concentrations were determined using BCA assay (Thermo Scientific). RIPA extracted brain lysates (20~ 50 μg) were run on 4–12% Bis-Tris gels (Invitrogen or Bio-Rad) with XT MES or XT MOPS running buffers. 3–8% Tris-acetate gels (Bio-Rad) with XT Tricine running buffer were used to separate and measure the ratio of cleaved N-terminal CHL1 fragment (CHL1_βNTF) to full-length CHL1 (CHL1_FL) in the hippocampus lysates, whereas 4–12% Bis-Tris gels (Bio-Rad) with XT MOPS running buffer were used to assess the CHL1 processing in the cortex homogenates. Proteins were transferred onto polyvinylidene difluoride (PVDF) membrane and blocked in 5% non-fat dried milk in TBST. The membrane was then incubated in the following antibodies: rabbit monoclonal anti-BACE1 (1:1500; D10E5; Cell signaling); rabbit polyclonal anti-GGA3 (1:1500; 4167; Cell signaling); rabbit polyclonal anti-GGA1 (1:2000; H-215; Santacruz); rabbit polyclonal anti-C-terminal APP antibody to detect mouse and human APP (1:5000; A8717; Sigma Aldrich); goat polyclonal anti-N-terminal CHL1 antibody to detect mouse CHL1_βNTF and CHL1_FL (1:1000; AF2147; R&D Systems); rat monoclonal anti-N-terminal CHL1 antibody to detect human CHL1_βNTF and CHL1_FL (1:2000; MAB2126; R&D Systems); mouse monoclonal anti-human APP antibody which recognizes the amino acid sequence 1–16 in Aβ region (1:10,000; 6E10; Biolegend); mouse monoclonal anti-calnexin to detect mouse and human calnexin (1:2000; 610523; BD biosciences); mouse monoclonal anti-GAPDH (1:10,000; MAP374; Millipore); and mouse monoclonal anti-β-tubulin (1:10,000; JDR.3BR; Sigma-Aldrich). Primary antibodies were detected with the species-specific HRP-conjugated secondary antibody, and then visualized by ECL. Chemiluminescent signal was captured on an LAS4000 Fuji Imager. Densitometry analysis was performed using Quantity One software (Bio-Rad).

### Aβ 42 ELISA

Brain PBS extracts were supplemented to reach a final concentration of 5 M guanidine HCl/50 mM Tris HCl and placed on a rotator overnight at 4 °C. Then, samples were diluted 1:20 in Reaction Buffer (Dulbecco’s phosphate buffered saline with 0.03% Tween-20 supplemented with 1X Protease inhibitor cocktail (Thermo Scientific). Samples were then centrifuged at 16,000×g for 20 min at 4 °C, and then the supernatants were collected. Final guanidine HCl concentrations were below 0.1 M. Protein concentrations were determined by BCA assay. The concentration of Aβ42 in Guanidine HCl extracts was measured by human Aβ 42 ELISA kits (KHB3441, Invitrogen) according to the manufacturer’s instruction. Optical signals at 450 nm were read on a Synergy 2 plate reader (Biotek) and sample concentrations were determined by comparison with the respective standard curves.

### Thioflavin-S staining

After perfusion with PBS, collected hemibrain for histological analysis was fixed in 4% paraformaldehyde in PBS for 24 h and cryoprotected in 30% sucrose in PBS containing 0.02% sodium azide. Coronal sections (40 μm) were cut on a sliding microtome. Every twelfth coronal sections were collected in cryoprotectant (30% sucrose, 1% Polyvinyl-pyrrolidone (PVP-40), 0.05 M phosphate buffer, 30% ethylene glycol), and then processed for histological analysis. Coronal sections from 4 and 12 months old male and female GGA3WT:5XFAD, GGA3Het;5XFAD, and GGA3KO;5XFAD mice were used to assess the effect of GGA3 deletion on amyloid plaques deposition by Thioflavin-S staining. First, free-floating coronal sections were washed three times with PBS. Sections were then treated with 0.1% Sudan Black in 70% ethyl alcohol for 20 min to quench non-specific autofluorescence. Samples were briefly differentiated two times in 70% ethanol, and then washed three times with PBS. Sections were stained with 0.025% Thioflavin-S in 50% ethanol for 8 min, and then differentiated two times with 50% ethanol. After three washes with PBS, sections were mounted on slides, and covered with mounting media. Fluorescent images were acquired on BZ-X700 all-in-one fluorescence microscope (Keyence). Captured images were used to analyze the size, number, and area of plaques in the hippocampus and cortex of male and female GGA3WT;5XFAD, GGA3Het;5XFAD, and GGA3KO;5XFAD mice with Fiji software (Image J).

### Quantification of images

Using a BZ-X700 all-in-one fluorescence microscope (Keyence), fluorescent images of 4 and 12 months old male and female GGA3WT;5XFAD, GGA3Het;5XFAD, and GGA3KO;5XFAD mice were acquired with 10X objective and then stitched using BZ-X analyzer Software. Stitched images (5–8 sections between bregma 0 and − 3.5 per mouse) were quantified using Fiji software (Image J). Captured stitched images were first converted to 8-bit black and white images. Then an intensity threshold (Triangle) for detecting the amyloid plaques area was applied to calculate the number, size, and percentage of total amyloid burden in the hippocampus or cortex.

### Serial fractionation of mouse brain samples

After weighing the snap frozen mouse hippocampi, samples were homogenized in 10 volumes of PBS buffer containing 1X protease inhibitor and 1X phosphatase inhibitor cocktail with 30 strokes on a Dounce homogenizer. About half of PBS homogenates were ultracentrifuged at 100,000×g for 1 h to obtain PBS fraction (soluble). The remaining pellet was washed with PBS buffer and spun at 100,000×g for 5 min. After removing the supernatant, remaining pellet was lysed in 10 volumes of RIPA buffer, sonicated, rotated for 30 min at 4 °C, and then ultracentrifuged at 100,000×g for 1 h. The supernatant was collected, and then stored in − 80 °C as RIPA fraction (membrane). Remaining half of PBS homogenates were mixed with 2X RIPA supplemented with protease and phosphatase inhibitor cocktail, sonicated and rotated for 30 min at 4 °C. After centrifugation at 14,000 xg for 20 min at 4 °C, the supernatant was collected as RIPA lysates (Total).

### Human brain samples

Frozen human hippocampus samples from subjects with Down syndrome (DS) or Unaffected Controls (Ctrls) were obtained from NIH NeuroBioBank (Table 1). Down syndrome samples were obtained from University of Maryland Brain and Tissue bank and unaffected control samples were obtained from University of Miami Brain Endowment Bank. All subjects are described in Table 1.

**Table 1:** Summary of sample Ages, Genders, and Postmortem intervals (PMI) of the human hippocampus

#	Diagnosis	Age	Gender	PMI
Down Syndrome				
1	DS	57	M	3
2	DS + AD	51	M	20
3	DS + AD	53	M	24
4	DS + AD	65	M	10
5	DS + AD	57	F	6
6	DS + AD	53	M	23
7	DS + AD	41	M	15
Unaffected Controls				
1	Ctrl	53	M	16
2	Ctrl	56	M	24
3	Ctrl	49	F	24
4	Ctrl	52	F	17
5	Ctrl	60	F	21
6	Ctrl	65	F	19
7	Ctrl	45	M	21
8	Ctrl	65	M	22

### Serial fractionation of human brain samples

For serial extraction of human samples, we used a modified sequential extraction method [12]. After weighing the frozen human hippocampus tissue, samples were homogenized in 10 volumes of ice-cold TBS buffer (50 mM Tris-HCL, 150 mM NaCl, pH 7.4) containing 1X protease inhibitor and 1X phosphatase inhibitor cocktail with 30 strokes on a Dounce homogenizer. About half of TBS homogenates was used for serial fractionation, the other half of TBS homogenates was used for RIPA extraction. About half of TBS homogenates was ultracentrifuged at 175,000×g for 30 min. The supernatant was collected, and then stored in − 80 °C as TBS fraction (soluble fraction). The remaining pellet was washed with TBS buffer and spun at 175,000×g for 5 min. After removing supernatant, the remaining pellet was resuspended in 10 volumes of TBS-TX buffer (1% Triton X-100 in TBS buffer containing 1X protease inhibitor and 1X phosphatase inhibitor cocktail), sonicated, rotated for 30 min at 4 °C, and then ultracentrifuged at 175,000×g for 30 min. The supernatant was collected, and then stored in − 80 °C as TBS-TX fraction (membrane fraction). The remaining half of TBS homogenates was mixed with 2X RIPA supplemented with protease and phosphatase inhibitor cocktail, sonicated and rotated for 30 min at 4 °C. After centrifugation at 14,000 xg for 20 min at 4 °C, the supernatant was collected as RIPA lysates (Total).

### Real-time quantitative PCR (RT-qPCR)

Total RNA was extracted from hippocampi of non-5XFAD and 5XFAD mice using RNeasy Mini kit (Quiagen) according to manufacturer’s instructions. Total RNA was analyzed using the Agilent 2100 Bioanalyzer to measure purity and integrity of RNA (Aligent Technologies). 1 μg of total RNA from each sample was subjected to reverse transcription reaction to synthetize cDNA using QuantiTect Reverse Transcription kit (Quiagen) according to manufacturer’s instruction. RT-qPCR was performed on an MX3000p qPCR System (Aligent Genomics) using the SYBR Green PCR Master Mix kit (Life Technologies). Primers were used to measure relative Chl1 gene expression levels (Primerbank ID: 110347544c3) normalized to the housekeeping gene Gapdh (Primerbank ID: 6679937a1). Relative expression levels were determined according to the ΔΔCt method when the expression level of the CHL1 mRNA is given by 2^-ΔΔCT^ where ΔΔCT = ΔCT CHL1 mRNA – ΔCT reference mRNA (Gapdh) in the same sample.

### Statistical analysis

Data are expressed as mean ± standard error of the mean (SEM), represented as error bars. One-way or two-way analysis of variance (ANOVA) with Fisher’s LSD post-hoc test was performed to evaluate statistical difference among the groups. Unpaired *t*-test with Welch’s correction was used for statistical comparison between means of amyloid burden in GGA3WT;5XFAD and GGA3KO;5XFAD mice. *P* < 0.05 was considered statistically significant for all experiments.

## Results

### BACE1 increases at similar levels in GGA3KO and GGA3KO;5XFAD mice at 4 months of age

**Fig. 1:**
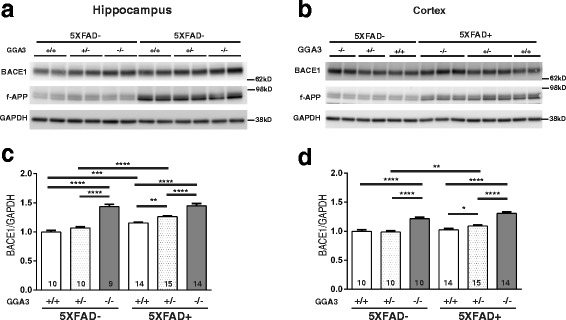
GGA3 deletion increases BACE1 at similar levels in young 5XFAD and non-5XFAD mice. **a-b** Representative immunoblots of hippocampus (**a**) and cortex (**b**) homogenates from 4 months old GGA3WT, GGA3Het, GGA3KO, GGA3WT;5XFAD, GGA3Het;5XFAD and GGA3KO;5XFAD mice probed with anti-BACE1 (D10E5), i-APP (A8717), and anti-GAPDH (MAB374) antibodies. Levels of full-length APP are increased in GGA3WT;5XFAD mice compared to GGA3WT mice **c-d** Densitometry levels of BACE1 were normalized to levels of GAPDH, which was used as loading control. Levels of BACE1 were expressed as relative to BACE1 levels in homogenates from GGA3WT mice set to 1.0. Graphs represent BACE1 levels in the hippocampus (**c**) or cortex (**d**) of mice from the six different genotypes. GGA3 deletion significantly increased BACE1 levels in the hippocampus and cortex from both 5XFAD and non-5XFAD mice. However, BACE1 levels in GGA3KO and GGA3KO;5XFAD mice were similar in both hippocampus and cortex. GGA3WT;5XFAD mice had significantly higher BACE1 levels than GGA3WT mice in the hippocampus but not in the cortex. BACE1 levels were also increased in both hippocampus and cortex of GGA3Het;5XFAD mice compared to GGA3Het mice. Moreover, GGA3 haploinsufficiency increased BACE1 levels in both the hippocampus and cortex of GGA3Het;5XFAD mice compared to GGA3WT;5XFAD mice. Total number of mice in each group is indicated within bars. All graphs represent mean ± SEM. One-way ANOVA with Fisher’s LSD post hoc tests was applied to each genotype group. * *p* < 0.05, ** *p* < 0.01, *** *p* < 0.001, **** *p* < 0.0001

It has been previously reported that levels of BACE1 increase with age in 5XFAD mice starting at the 6 months of age [13]. Given that GGA3 deletion increases cerebral levels of BACE1 in mice [10], we hypothesized that GGA3 deletion would lead to BACE1 elevation in 5XFAD mice at an earlier age (e.g. 4 months of age). Thus, we first analyzed the levels of BACE1 in the hippocampus and cortex of 4 months old of GGA3WT, GGA3Het, GGA3KO, GGA3WT;5XFAD, GGA3Het;5XFAD, and GGA3KO;5XFAD mice (Fig. 1a and b). We found that BACE1 levels were increased in the hippocampus of GGA3WT;5XFAD mice compared to GGA3WT mice. As we have previously reported, BACE1 levels were increased both in the hippocampus and cortex of GGA3KO mice compared to GGA3WT mice (Fig. 1c and d). However, levels of BACE1 were similar in GGA3KO and GGA3KO;5XFAD mice (Fig. 1c and d). Interestingly, GGA3 haploinsufficiency potentiates BACE1 elevation both in the hippocampus and cortex of 5XFAD mice. Levels of BACE1 were increased both in the hippocampus and the cortex of GGA3Het;5XFAD compared to GGA3WT;5XFAD mice, and GGA3Het;5XFAD compared to GGA3Het mice (Fig. 1c and d). Two-way ANOVA showed no significant difference in BACE1 levels between sex in hippocampus (F(1, 60) = 2.12, *p* = 0.15) and cortex samples (F(1, 61) = 0.043, *p* = 0.83), thus the data from both sexes were combined. Taken together, these data indicate that while 5XFAD mutations did not further increase BACE1 levels when GGA3 is deleted, GGA3 haploinsufficiency potentiated BACE1 elevation in 4 months old 5XFAD mice.

### GGA3 levels are reduced in both hippocampus and cortex of 5XFAD mice at 4 months of age

**Fig. 2:**
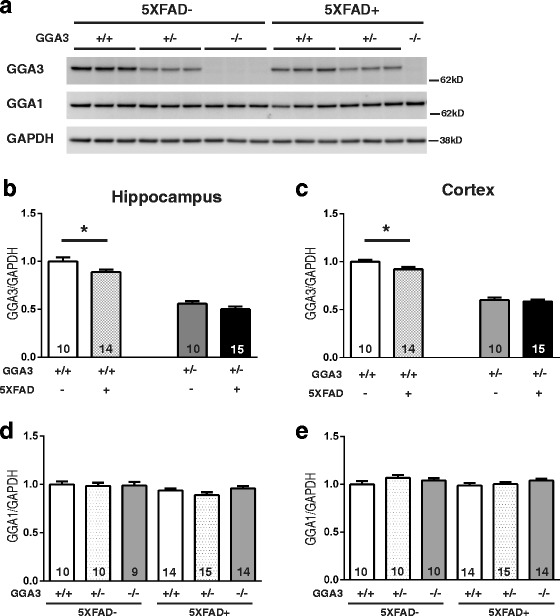
GGA3 levels are reduced in both hippocampus and cortex of young 5XFAD mice. **a** Representative immunoblot of cortex homogenates from 4 months old GGA3WT, GGA3Het, GGA3KO, GGA3WT;5XFAD, GGA3Het;5XFAD and GGA3KO;5XFAD mice using anti-GGA3 (4167), anti-GGA1 (H-215), anti-GAPDH (MAB374) antibodies. **b-c** Graphs represent densitometry levels of GGA3 normalized to levels of GAPDH in hippocampus (**b**) and cortex (**c**) homogenates form each genotype, expressed as relative value to GGA3 levels in GGA3WT mice set to 1.0. Levels of GGA3 were reduced in both hippocampus and cortex of 4 months old GGA3WT;5XFAD mice compared to GGA3WT mice. **d-e** Graphs represent GGA1 levels normalized to levels of GAPDH in hippocampus (**d**) and cortex (**e**) homogenates form each genotype, expressed as relative value of GGA1 levels in GGA3WT mice set to 1.0. No changes in the levels of GGA1 were observed in all genotypes. Total number of mice in each group is indicated within bars. All graphs represent mean ± SEM. One-way ANOVA with Fisher’s LSD post hoc tests was applied to each genotype group. * *p* < 0.05

We have previously shown that that levels of GGA3 are decreased and inversely correlated with BACE1 levels in post-mortem AD brains [8]. Our original findings have been confirmed by two independent studies conducted in AD brain samples of Australian and European origin [14] (US Patent Application 20120276076, Annaert, Wim; et al. November 1, 2012). More recently, we have reported that not only GGA3 but also its homologue GGA1 is a substrate for caspase-3. Accordingly, we found that not only GGA3 but also GGA1 was depleted in postmortem AD brains with elevated BACE1 levels [10]. Thus, we measured GGA3 and GGA1 levels in the hippocampus and cortex of mice from the six different genotypes at 4 months of age (Fig. 2a). We found that GGA3 levels were decreased in both hippocampus and cortex of GGA3WT;5XFAD mice compared to GGA3WT littermates (Fig. 2b and c). In contrast, levels of GGA1 were similar in all genotypes (Fig. 2d and e). Given that Aβ pathology is still at early stage in 5XFAD mice at 4 months of age, these data suggest that the GGA1 depletion observed in AD brains may be a late event in the progression of the disease. No difference was found between sexes for GGA3 and GGA1 levels in the hippocampus (Two-way ANOVA, F(1, 41) = 0.24, *p* = 0.63 and F(1, 59) = 2.98, *p* = 0.90, respectively) and cortex (F(1, 41) = 1.69, *p* = 0.20 and F(1, 61) = 1.38, *p* = 0.24, respectively), thus, data from male and female mice were combined. These data collectively suggest that AD pathology leads to depletion of GGA3 not only in human brains but also in a mouse model of AD. Thus, 5XFAD mice represent a good model to study the impact of GGA3 depletion on AD pathology and in particular BACE1 accumulation.

### GGA3 deletion does not increase levels of Aβ42 and amyloid plaques in 5XFAD mice at 4 months of age

**Fig. 3:**
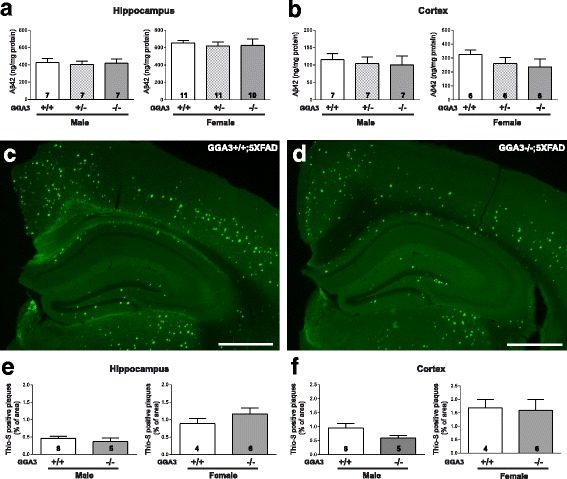
GGA3 deletion does not increase levels of Aβ42 and amyloid plaques in young 5XFAD mice. **a-b** Human Aβ42 levels were measured by ELISA (ng/mg of total protein) (Invitrogen) in hippocampus (**a**) and cortex (**b**) GuHCl extracts from of 4 months old GGA3WT;5XFAD, GGA3Het;5XFAD and GGA3KO;5XFAD male and female mice. Levels of Aβ42 were similar in all genotypes at 4 months of age. Total number of mice analyzed is indicated within bars. One-way ANOVA with Fisher’s LSD post hoc tests was applied to each genotype group. **c-d** Coronal brain sections from 4 months old GGA3WT;5XFAD and GGA3KO;5XFAD male and female mice were used to analyze amyloid plaques by Thioflavin-S staining. Representative images show amyloid plaques from 4 months old female GGA3WT;5XFAD (**c**) and GGA3KO;5XFAD (**d**) mice. Scale bar is 500 μm. **e-f** The graphs represent the percentage area occupied by amyloid plaques stained with Thioflavin-S in hippocampus (**e**) and cortex (**f**) of GGA3WT;5XFAD and GGA3KO;5XFAD male and female mice at the age of 4 months. GGA3 deletion does not affect amyloid burden in 4 months old 5XFAD mice. Total number of mice in each group is indicated within bars. All graphs represent mean ± SEM. Unpaired t-test with Welch’s correction was performed

Given that BACE1 levels were increased in the GGA3KO;5XFAD mice compared to GGA3WT;5XFAD mice, we next determined whether GGA3 deletion leads to an increase in levels of Aβ42 and amyloid plaques. Guanidine hydrochloride (GuHCl) protein extracts of hippocampus and cortex of 4 months old GGA3WT;5XFAD, GGA3Het;5XFAD and GGA3KO;5XFAD mice were analyzed by ELISA (Invitrogen) to measure Aβ42 levels. Since female 5XFAD mice have higher Aβ42 levels than male 5XFAD mice [11, 15], we analyzed Aβ42 levels in males and females separately. In spite of the increase in BACE1 levels in GGA3KO;5XFAD mice compared to GGA3WT;5XFAD mice (Fig. 1), Aβ42 levels were similar in all genotypes in both male and female mice (Fig. 3a-b and Additional file 1). We furthered our experiment by measuring the amyloid burden by Thioflavin-S staining in the hippocampus and cortex of GGA3WT;5XFAD, GGA3Het;5XFAD and GGA3KO;5XFAD mice. We found no difference among different genotypes at 4 months of age in both male and female mice (Fig. 3c-f). Accordingly, we found that levels of BACE1-derived APP-CTFs (phosphoC99, C99, and phosphoC89) were similar among the different genotypes (Additional file 2).

### GGA3 deletion increases BACE1-mediated cleavage of CHL1 in the hippocampus of 5XFAD and non-5XFAD mice at 4 months of age

In addition to APP, more than 40 BACE1 substrates have been identified by quantitative proteomics analysis [16–18] and some of them have been validated in vitro and in vivo (for review see [19, 20]). The neural cell adhesion molecule L1-like protein (CHL1), which is required for axonal targeting, has been shown to be a BACE1 substrate [21, 22]. Given that elevated BACE1 did not increase APP processing in GGA3KO;5XFAD mice at 4 months of age, we next investigated the effect of GGA3 deletion and consequent BACE1 elevation on the processing of endogenous CHL1.

**Fig. 4:**
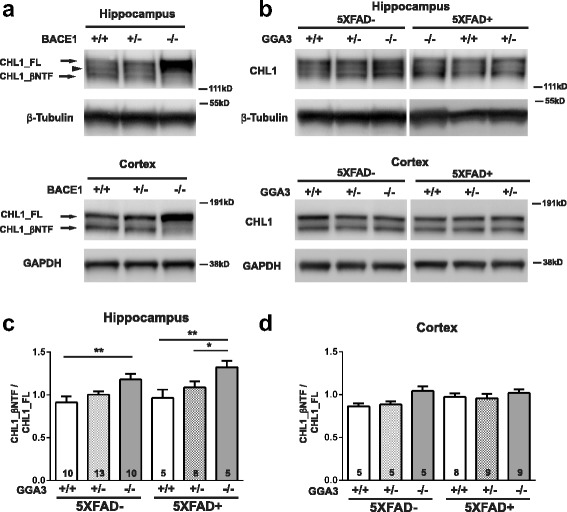
BACE1-mediated cleavage of CHL1 is increased in the hippocampus of young GGA3KO and 5XFAD;GGA3KO mice. **a** Representative immunoblots of hippocampus (top) and cortex (bottom) homogenates from BACE1+/+, BACE1+/−, and BACE1−/− mice using anti-N-terminal CHL1 antibody (AF2147). Brain homogenates with reduced BACE1 exhibit increased intensity of full-length CHL1 (CHL1_FL) at ~ 185 kDa and a reduction (BACE1+/−) or absence (BACE1−/−) of BACE1-cleaved N-terminal CHL1 (CHL1_βNTF) at ~ 165 kDa. An ~ 175 kDa band between CHL1_FL and CHL1_βNTF may present an alternative splicing isoform or glycosylated CHL1 in hippocampus. **b** Representative immunoblots of CHL1_FL and CHL1_βNTF levels in hippocampus (top) and cortex (bottom) homogenates from 4 months old GGA3WT, GGA3Het, GGA3KO, GGA3WT;5XFAD, GGA3Het;5XFAD and GGA3KO;5XFAD mice. **c-d** Graphs represent the ratio of CHL1_βNTF to CHL1_FL in hippocampus (**c**) and cortex (**d**) homogenates in the six different genotypes. GGA3 deletion significantly increased CHL1_βNTF/CHL1_FL ratio in the hippocampus of non-5XFAD and 5XFAD mice, whereas no changes were observed in the cortex. All graphs represent mean ± SEM. One-way ANOVA with Fisher’s LSD post hoc tests was applied to each genotype group. * *p* < 0.05, ** *p* < 0.01

We first confirmed that CHL1 is a BACE1 substrate by analyzing its processing in the hippocampus and cortex homogenates from BACE1WT, BACEHet, and BACE1KO mice. In agreement with a previous study [21], in the adult hippocampus 3 bands were detected: CHL1_FL (185 kDa), CHL1_βNTF (~ 165 kDa) and a band of ~ 175 kDa that most likely is an alternative splicing isoform or glycosylated CHL1. Western blot analysis using anti-N-terminal CHL1 antibody (AF2147) revealed an increase in full-length CHL1 (CHL1_FL) and significantly reduced N-terminal CHL1 fragment (CHL1_βNTF) in both hippocampus and cortex of BACE1KO mice compared to BACE+/+ mice. A slight increase in CHL1_FL and decrease in CHL1_βNTF were observed in both hippocampus and cortex of BACE+/− mice compared with BACE1WT mice (Fig. 4a). Next, the ratio of CHL1_βNTF to CHL1_FL was measured by immunoblot analysis in the hippocampus and cortex lysates from mice of the six different genotypes at 4 months of age (Fig. 4b). Since there was no sex difference in BACE1 levels among all genotypes, data from male and female mice were combined. BACE1-mediated CHL1 cleavage was increased by 29.4% (*p* = 0.003) in the hippocampus of GGA3KO mice compared to GGA3WT mice. Similarly, the ratio of CHL1_βNTF to CHL1_FL was increased by 37.6% (*p* = 0.02) in hippocampus of GGA3KO;5XFAD mice compared to GGA3WT;5XFAD mice (Fig. 4c and d). In contrast, BACE1-mediated processing of CHL1 was similar among all genotype in the cortex. A possible explanation for the increased CHL1 processing observed in the hippocampus and not in the cortex of GGA3KO mice is that CHL1 is predominantly expressed in the subiculum, mossy fiber bundles, inner molecular layer of dentate gyrus, and stratum oriens [23]. Similarly, BACE1 is highly expressed in the mossy fiber bundles [21, 24]. Accordingly, axonal guidance defects are observed in the hippocampal mossy fibers of both CHL1−/− and BACE1−/− mice [21, 23].

Collectively, these data indicate that the BACE1 elevation engendered by GGA3 deletion was sufficient to increase CHL1 but not APP processing in both 5XFAD and non-5XFAD mice, most likely because BACE1 preferentially cleaves CHL1 over APP.

### GGA3 deletion prevents age-dependent elevation of BACE1 in 5XFAD mice

**Fig. 5:**
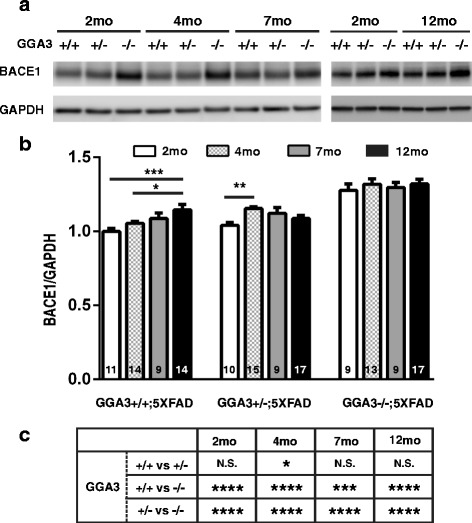
GGA3 deletion prevents age-dependent elevation of BACE1 in 5XFAD mice. **a** Representative immunoblots of hippocampus homogenates from 2, 4, 7 and 12 months old GGA3WT;5XFAD, GGA3Het;5XFAD and GGA3KO;5XFAD mice probed with anti-BACE1 (D10E5), and anti-GAPDH (MAB374) antibodies. **b** BACE1 levels normalized to GAPDH in hippocampus from 5XFAD mice at 2, 4, 7, and 12 months of age are presented relative to the levels of BACE1 in homogenates from 2 months old GGA3WT;5XFAD mice (set to 1.0). 12 months old GGA3WT;5XFAD mice have significantly higher BACE1 levels than 2 months old GGA3WT;5XFAD mice. However, age-dependent elevation of BACE1 was not observed in 12 months old GGA3KO;5XFAD mice compared to 2 months old GGA3KO;5XFAD mice. **c** The table shows statistical analysis of BACE1 levels in hippocampus of GGA3WT;5XFAD, GGA3Het;5XFAD and GGA3KO;5XFAD mice within the same age group. Total number of mice in each group is indicated within bars. All graphs represent mean ± SEM. Two-way ANOVA with Fisher’s LSD post hoc tests was applied to each genotype group. * *p* < 0.05, ** *p* < 0.01, *** *p* < 0.001, **** p < 0.0001

Levels of BACE1 increase with age in 5XFAD mice starting at the age of 6 months [13]. Thus, we investigated whether GGA3 haploinsufficiency or deletion affects the age-dependent BACE1 elevation in the 5XFAD mice. Levels of BACE1 were quantified in the hippocampus homogenates from GGA3WT;5XFAD, GGA3Het;5XFAD, and GGA3KO;5XFAD mice at 2, 4, 7 and 12 months of age (Fig. 5a and b). BACE1 elevation was observed in the hippocampus homogenates of 12 months old GGA3WT;5XFAD mice compared to 2 and 4 months old GGA3WT;5XFAD mice. Also, BACE1 levels were increased in the hippocampus of 4 months old GGA3Het;5XFAD mice compared to 2 months old GGA3Het;5XFAD mice. However, the levels of BACE1 were similar in the hippocampus of GGA3KO;5XFAD mice at all ages (Fig. 5a and b). As expected, BACE1 levels were significantly increased in the hippocampus of GGA3KO;5XFAD mice compared to GGA3WT;5XFAD mice or to GGA3Het;5XFAD of same age (Fig. 5c). Since Two-way ANOVA showed no significant difference in BACE1 levels between males and females at 2, 4, 7 and 12 months of age in hippocampus (F(1, 24) = 2.6, *p* = 0.12; F(1, 36) = 0.11, *p* = 0.74; F(1, 21) = 0.13, *p* = 0.73; F(1, 42) = 0.30, *p* = 0.59, respectively) samples, the data from both sexes were combined. We did not observe a significant increase in BACE1 levels between 2 and 12 months old GGA3KO;5XFAD mice, thus these data indicate that GGA3 deletion prevents age-dependent elevation of BACE1 in 5XFAD mice.

### GGA3 levels decrease in hippocampus of 5XFAD mice with age

**Fig. 6:**
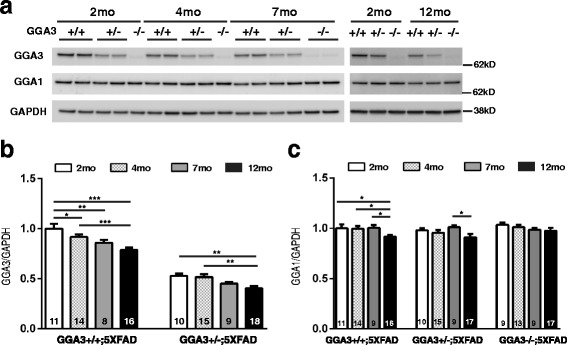
GGA3 levels are reduced in the hippocampus of 5XFAD mice with age. **a** Representative immunoblot of hippocampus homogenates from 2, 4, 7 and 12 months old GGA3WT;5XFAD, GGA3Het;5XFAD and GGA3KO;5XFAD mice probecr27d with anti-GGA3 (4167), anti-GGA1 (H-215), anti-GAPDH (MAB374) antibodies. **b-c** GGA3 and GGA1 levels, normalized to GAPDH in the hippocampus homogenates of GGA3WT;5XFAD and GGA3Het;5XFAD mice at 2, 4, 7 and 12 months of age, are expressed as relative values to the levels of GGA3 in 2 months old GGA3WT;5XFAD homogenates set to 1.0. Levels of GGA3 decrease in both GGA3WT;5XFAD and GGA3Het;5XFAD mice with age. In contrast, levels of GGA1 were decreased only in 12 months old GGA3WT;5XFAD mice compared to 2 months old GGA3WT;5XFAD mice. Total number of mice in each group is indicated within bars. All graphs represent mean ± SEM. Two-way ANOVA with Fisher’s LSD post hoc tests was applied to each genotype group. * *p* < 0.05, ** *p* < 0.01

As we observed the age-dependent BACE1 elevation in 5XFAD mice, we went on to investigate if levels of GGA3 and GGA1, two proteins that negatively correlated with elevated BACE1 in AD brains, reduce with age, and could be an underlying mechanism of BACE1 accumulation in aging brains. Hippocampal levels of GGA3 in GGA3WT;5XFAD and GGA3Het;5XFAD mice were measured at four different ages (2, 4, 7 and 12 months) using immunoblot analysis (Fig. 6a). We found that GGA3 levels were significantly decreased in the hippocampus of 4, 7 and 12 months old GGA3WT;5XFAD mice compared to 2 months old GGA3WT;5XFAD mice (Fig. 6b). Moreover, GGA3 levels were reduced in the hippocampus, in 12 months old GGA3Het;5XFAD mice compared to 2 and 4 months old GGA3Het;5XFAD mice (Fig. 6b). In contrast, levels of GGA1 were decreased only in 12 months old GGA3WT;5XFAD mice compared to 2 months old GGA3WT;5XFAD mice, suggesting that GGA1 depletion is associated with more advanced pathology (Fig. 6c). Since two-way ANOVA showed no significant difference in GGA3 levels between males and females at 2, 4, 7 and 12 months of age in hippocampus (F(1, 17) = 0.51, *p* = 0.49, F(1, 25) = 0.005, *p* = 0.94; F(1, 14) = 0.24, *p* = 0.64, F(1, 28) = 0.21, *p* = 0.65 respectively) samples, the data from both sexes were combined. These results indicate that the amount of GGA3 in the hippocampus decreases while BACE1 increases with age in 5XFAD mice, similar to what is observed in human AD brains, and suggest that GGA3 depletion is a leading candidate mechanism underlying BACE1 accumulation in AD.

### GGA3 deletion increases levels of Aβ42 and amyloid plaques in the hippocampus of 5XFAD mice at 12 months of age

**Fig. 7:**
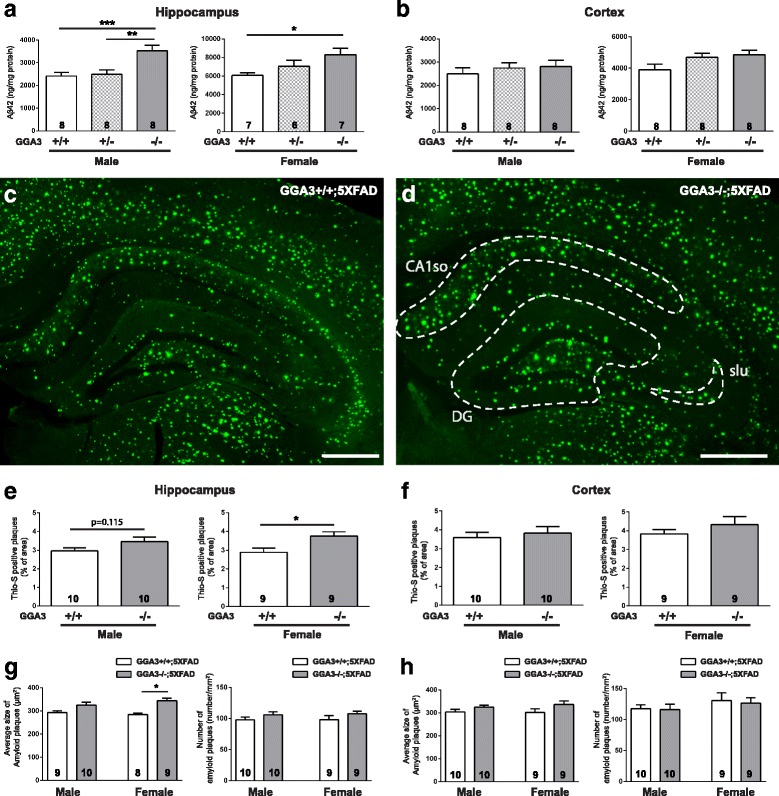
GGA3 deletion increases levels of Aβ42 and amyloid plaques increase in the hippocampus of old 5XFAD;GGA3KO mice at 12 months of age. **a**-**b** Levels of human Aβ42 were quantified by ELISA (ng/mg of total protein) (Invitrogen) in hippocampus (**a**) and cortex (**b**) GuHCl extracts from 12 months old GGA3WT;5XFAD, GGA3Het;5XFAD and GGA3KO;5XFAD male and female mice. GGA3 deletion resulted in increased Aβ42 levels in the hippocampus of both male and female 12 months old 5XFAD mice. GGA3KO;5XFAD mice also had a trend of increased Aβ42 levels in the cortex at 12 months of age. One-way ANOVA with Fisher’s LSD post hoc tests was applied to each genotype group. * p < 0.05, ** < 0.01, *** *p* < 0.001. **c-d** Coronal brain sections from 12 months old GGA3WT;5XFAD and GGA3KO;5XFAD male and female mice were stained with Thioflavin-S. Representative images show amyloid plaques from 12 months old female GGA3WT;5XFAD (**c**) and GGA3KO;5XFAD mice (**d**). Increased Thioflavin-S positive amyloid plaques were frequently observed in the area of dentate gyrus (DG), stratum lucidum (slu), and CA1 stratum oriens (CA1so) at 12 months of age (**d**). Scale bar is 500 μm. **e-h** The graphs represent the quantification of Thioflavin-S positive plaques in hippocampus (**e**) and cortex (**f**) of GGA3WT;5XFAD and GGA3KO;5XFAD male and female mice. GGA3KO;5XFAD female mice have significantly increased amyloid plaque burden in hippocampus, but not in cortex, compared to GGA3WT;5XFAD females at 12 months of age. GGA3KO;5XFAD male mice also show a similar trend when compared to GGA3WT;5XFAD males. **g-h** The graphs show average size of amyloid plaques (μm^2^) and number of amyloid plaques in hippocampus and cortex of GGA3WT;5XFAD and GGA3KO;5XFAD mice. A significantly increase in size of amyloid plaques but not number of amyloid plaques was found in hippocampus of GGA3KO;5XFAD females. Total number of mice in each group is indicated within bars. All graphs represent mean ± SEM. Unpaired t-test with Welch’s correction was performed. * p < 0.05, ** p < 0.01, *** p < 0.001

To further assess the effect of GGA3 deletion on AD pathology in aged brain, we next analyzed levels of Aβ42 by ELISA in 12 months old GGA3WT;5XFAD, GGA3Het;5XFAD and GGA3KO;5XFAD mice. We found that levels of Aβ42 were increased in the hippocampus of both male and female GGA3KO;5XFAD mice compared to GGA3WT;5XFAD mice (Fig. 7a and Additional file 3). In contrast, levels of Aβ42 in the cortex of all genotypes were similar in both sexes (Fig. 7b and Additional file 3).

We furthered our experiment by measuring the amyloid burden by Thioflavin-S staining in the hippocampus and cortex of GGA3WT;5XFAD and GGA3KO;5XFAD mice. We found that amyloid burden was increased in the hippocampus but not the cortex of 12 months old GGA3KO;5XFAD females compared to GGA3WT;5XFAD females (Fig. 7c-f and Additional file 3). Accumulation of Thioflavin-S positive amyloid plaques were more frequently observed in dentate gyrus (DG), stratum lucidum (slu), and CA1 stratum oriens (CA1so) of GGA3KO;5XFAD females compared to GGA3WT;5XFAD females at 12 months of age (Fig. 7c-d). A trend of increased amyloid burden was observed in the hippocampus of GGA3KO;5XFAD males, but it did not reach a statistical difference when compared to GGA3WT;5XFAD males (Fig. 7e). A further analysis revealed that the increase in amyloid burden in the hippocampus of 12 months old female GGA3KO;5XFAD mice compared to GGA3WT;5XFAD littermates was due to an increase in the plaque size rather than the number of plaques (Fig. 7g and h).

### Increased amyloid burden is associated with increased levels of transgenic APP in female 5XFAD mice at 12 months of age

Given that levels of BACE1 do not increase in GGAKO;5XFAD mice with age (Fig. 5), we eliminate a mechanism based on the effects of the enzymatic function that generates Aβ. Next, we wonder if differential expression of the BACE1 substrate, namely APP, could be the underlying reason for our findings in Aβ42 levels and amyloid burden. The fact that sexual difference in Thioflavin-S staining was observed, consistent with sex-difference in APP expression in 5XFAD mice that has previously described [15], makes investigating APP a logical next step. Therefore, we next analyzed transgenic human APP levels using anti-human APP antibody (6E10).

**Fig. 8:**
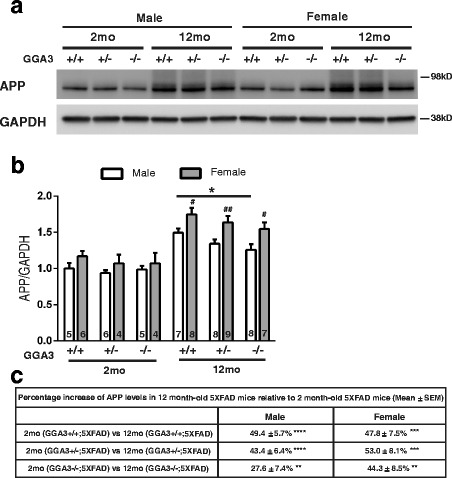
Increased amyloid burden is associated with increased levels of APP in old female 5XFAD mice. **a** Representative immunoblot of hippocampus homogenates from 2 and 12 months old GGA3WT;5XFAD, GGA3Het;5XFAD, and GGA3KO;5XFAD male and female mice probed with anti-human APP (6E10) and anti-GAPDH (MAB374) antibodies. **b** Densitometry levels of transgenic human APP were quantified, normalized to GAPDH, and then expressed as relative values to their levels in 2 months old GGA3WT;5XFAD homogenates set to 1.0. For all GGA3 genotypes, 5XFAD females had significantly higher levels of APP compared to 5XFAD males at 12 months of age, but not 2 months of age (# p < 0.05, ## p < 0.01). GGA3KO;5XFAD males had reduced levels of APP compared to GGA3WT;5XFAD males (* p < 0.05). **c** The table shows summary of percentage increase of APP levels in 12 months old mice relative to genotype-matched 2 months old mice. ** p < 0.01, *** p < 0.001, **** *p* < 0.0001. Two-way ANOVA with Fisher’s LSD post hoc tests was applied to each sex or genotype group

We found that levels of transgenic APP were higher in 12 months old mice compared to 2 months old mice with the same genotype and sex (Fig. 8a and c). These findings were confirmed in another APP transgenic line (APPswe/PS1∆E9) (Additional file 4). Moreover, levels of transgenic human APP were higher in 12 months old females compared to males within the same genotype of 5XFAD mice (Fig. 8b). Interestingly, levels of APP were decreased in GGA3KO;5XFAD males compared to GGA3WT;5XFAD males. GGA3KO;5XFAD females also showed a similar trend toward decreased APP levels compared to GGA3WT;5XFAD females. We speculate that increased BACE1 levels via GGA3 deletion might result in increased APP processing, and decreased APP levels were consequently observed in GGA3KO;5XFAD mice at 12 months of age. We also analyzed APP-CTFs and found that levels of BACE1-derived APP-CTFs (phosphoC99, C99, and phosphoC89) were similar among the different genotypes (Additional file 5). Given that BACE1 elevation engendered by GGA3 deletion is ~ 30% compared to GGA3WT;5XFAD mice, we were not surprised to find no changes in levels of phospho-C99, C99, and phospho-C89 fragments in 12 months old GGAWT;5XFAD, GGA3Het;5XFAD and GGA3KO;5XFAD mice. Similarly Sadleir et al. [15] found that Aβ42 levels and amyloid burden decreased in BACE1Het;5XFAD female mice. While levels of APP were increased no significant changes in APP-C99 and sAPPβ were observed at 6 and 9 months of age in BACE1Het;5XFAD female mice. Furthermore, McConlogue et al. [25] reported a decrease in Aβ42 levels but no change in sAPPβ in 13 month-old BACE1Het;PDAPP mice. All together, these data indicate that increased Aβ42 levels and amyloid burden due to GGA3 deletion in 5XFAD mice, an effect that is more profound in females, is associated with elevated levels of transgenic APP.

### BACE1-mediated cleavage of CHL1 is reduced in 12 months old 5XFAD mice, and the decrease is much more pronounced in female than male 5XFAD mice

In 4 months old 5XFAD mice, we found that elevated BACE1 due to GGA3 deletion increased BACE1-mediated cleavage of CHL1 (ratio of CHL1_βNTF to CHL1_FL) without affecting the generation of amyloid beta. In contrast, Aβ42 levels were increased in 12 months old GGA3KO;5XFAD mice compared to GGA3WT;5XFAD mice, most likely owing to elevated transgenic human APP levels. These findings raised the question of whether the elevation of APP and its increased processing could affect the cleavage of CHL1 at 12 months of age.

**Fig. 9:**
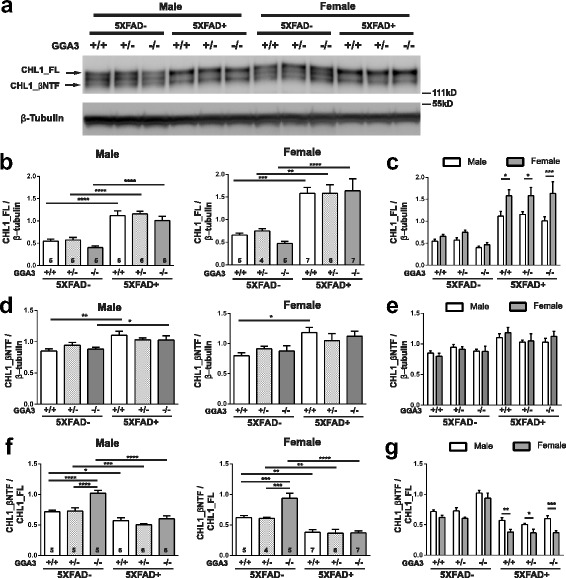
BACE1-mediated cleavage of CHL1 is reduced in old 5XFAD mice. **a** Representative immunoblot of hippocampus homogenates from 12 months old GGA3WT, GGA3Het, GGA3KO, GGA3WT;5XFAD, GGA3Het;5XFAD, and GGA3KO;5XFAD male and female mice probed with anti-N-terminal CHL1 antibody (AF2147) and anti-β-tubulin (JDR.3B8). **b-c** The graphs represent CHL1_FL levels normalized to the levels of β-tubulin in samples from six different genotypes of male and female mice separately. Among all GGA3 genotypes, CHL1_FL levels were significantly increased in both male and female 5XFAD mice compared to gender-matched non-5XFAD mice, and such increase was more pronounced in females than males (**c**). **d-e** The graphs represent levels of CHL1_βNTF/β-tubulin levels normalized to the levels of β-tubulin in samples from six different genotypes of male and female mice separately. Both male and female 5XFAD mice have increased CHL1_ β NTF levels compared to non-5XFAD mice. However, CHL1_β NTF levels were similar in both males and females (**e**). **f-g** The graphs represent the ratio of CHL1_βNTF to CHL1_FL in the six different genotypes of male and female mice separately. The CHL1_βNTF/ CHL1_FL ratio was increased in both male and female GGA3KO mice compared to sex-matched GGA3WT littermates. In contrast, the CHL1_βNTF/CHL1_FL ratio was significantly decreased in 5XFAD mice compared to genotype-matched non-5XFAD mice and such decrease was greater in females than males (**g**). Total number of mice in each group is indicated within bars. All graphs represent mean ± SEM. One-way (**b**, **d**, and **f**) or Two-way ANOVA (**c**, **e**, and **g**) with Fisher’s LSD post hoc tests were applied to each sex or genotype group. * p < 0.05, ** p < 0.01, *** p < 0.001, **** < 0.0001

First we measured the levels of CHL1_FL and CHL1_βNTF in the hippocampus homogenates of mice from the six genotypes. Male and female mice were analyzed separately. CHL1_FL levels were slightly decreased in both male and female GGA3KO mice compared to wild type mice, consistent with an increased BACE1-mediated processing. In contrast, CHL1_FL levels were dramatically increased in 5XFAD mice compared to GGA3 genotype-matched non-5XFAD mice, similar to the increased CHL1_FL levels observed in BACE1KO mice, and such increase was more pronounced in females than males (Figs. 4a and 9a-c). While levels of CHL1_βNTF were similar across genotypes in non-5XFAD mice, they were increased in both male and female 5XFAD mice compared to non-5XFAD mice (Fig. 9d). In spite of such elevation, the CHL1_βNTF/CHL1_FL ratio was significantly decreased in 5XFAD mice compared to genotype-matched non-5XFAD mice and such decrease was greater in females than males (Fig. 9f-g). We also found that the ratio of CHL1_βNTF to CHL1_FL was increased in 12 months old GGA3KO mice compared to GGA3WT mice (Fig. 9a and f), similar to the observation in 4 months old mice and consistent with increased levels of BACE1 (Fig. 4).

In order to further confirm our data and better characterize CHL1 processing, we carried out serial fractionation of the hippocampus from BACE1WT, BACEHet and BACEKO mice to separate soluble and membrane fractions. Western blot analysis of CHL1 clearly detected two membrane bound CHL1 fragments in the membrane fraction (Additional file 6), corresponding to CHL1_FL (~ 185 kDa) and the ~ 175 kDa band (arrowhead) also detected in the total extract (Figs. 4b and 9a). CHL1_FL levels were increased in BACE1KO in the membrane fraction and total extract as shown in Fig. 4a. CHL1_βNTF was detected in the soluble fraction and corresponds to the ~ 165 kDa band detected in the total extract (Additional file 6). As previously described [26], a band of slightly lower molecular weight (indicated by an asterisk) was detected in the soluble fraction from BACE1KO mice. Such soluble fragment is most likely derived by a compensatory increased cleavage of CHL1 by ADAM8 or ADAM10 [27, 28].

Next, we prepared hippocampal fractions from 12 months old GGA3WT, GGAKO, BACE1Het, BACE1KO, 5XFAD, and BACE1Het;5XFAD mice. Samples from BACE1KO were included as negative control for BACE1-mediated processing of CHL1. BACE1Het and BACE1Het;5XFAD mice were analyzed to assess the effect of 50% reduction of BACE1 on levels of CHL1_FL and CHL1_βNTF.

Western blot analysis revealed that levels of CHL1_FL were decreased in the membrane fraction while CHL1_βNTF levels were increased in the soluble fraction of GGAKO compared to GGA3WT mice (Additional file 7A–B). As a result the ratio CHL1_βNTF/CHL1_FL was increased, consistent with an increased BACE1-mediated processing of CHL1 in GGAKO mice as reported in Figs. 4c and 9f. In contrast, levels of CHL1_FL were significantly increased in the membrane fraction from BACE1Het compared to wild type mice (indicated as GGA3+/+) and in BACE1Het;5XFAD compared to BACE1Het and 5XFAD mice. However, levels of soluble CHL1_βNTF were not significantly reduced in the soluble fraction of BACE1Het and BACE1Het;5XFAD compared to control (GGA3WT and 5XFAD mice, respectively). Furthermore, the ratio CHL1_βNTF/CHL1_FL was reduced in BACE1Het and BACE1Het;5XFAD but not reach a significant difference when compared to control. These findings indicate that a 50% decrease of BACE1-mediated processing leads to an accumulation of CHL1_FL in absence of a reduction of CHL1_βNTF. Similarly, levels of CHL1_FL were significantly increased in the membrane fraction from 5XFAD mice compared to wild type mice (indicated as GGA3+/+) as reported in Fig. 9a and b. Levels of CHL1_βNTF were increased in the soluble fraction as well as in the total extract of 5XFAD mice compared to wild type (Additional file 7A–B and Fig. 9d). As observed in BACE1Het mice, the ratio CHL1_βNTF/CHL1_FL was reduced in the 5XFAD but did not reach a significant difference when compared to control most likely due to the fact that soluble fragments are detected even in absence of BACE1 in the soluble fraction (see BACE1KO fractionation). Another possible explanation is that while in the total extract CHL1_βNTF/CHL1_FL ratio was performed within the same sample, in the soluble and membrane fractions fragments are normalized against loading controls specific for each fraction.

To rule out the possibility that the increase in CHL1_FL observed in 5XFAD mice was due to increased expression we assessed the mRNA levels of Chl1 in hippocampi from both non-5XFAD and 5XFAD mice by RT-qPCR. The levels of Chl1 mRNA were similar in the hippocampus of 5XFAD mice compared with non-5XFAD mice (Additional file 8A). Furthermore, Chl1 expression was similar in 5XFAD males and females in spite of the increased levels of CHL1_FL observed in 5XFAD females compared to 5XFAD males (Additional file 8B and Fig. 9c). Altogether, these results indicate that CHL1 expression is not affected by transgenic APP expression in 5XFAD mice. An alternative explanation is that both reduced BACE1-cleavage and protein stabilization associated with aging result in increased levels of CHL1_FL and CHL1_βNTF.

These data suggest that the elevated transgenic APP might compete with CHL1 for BACE1 processing, resulting in the accumulation of full-length CHL1 in 12 months old 5XFAD mice. Such speculation was further confirmed with the observation that levels of CHL1_FL were significantly higher in female 5XFAD mice compared to males with matching genotypes (Fig. 9b). Since female 5XFAD mice have higher levels of transgenic APP than males with corresponding genotype (Fig. 8e), the competition of APP over CHL1 on BACE1 is furthered in female 5XFAD mice, resulting in a sexually differential stabilization of CHL1_FL.

### BACE1-mediated cleavage of CHL1 is reduced in human brains from subjects affected by Down syndrome

**Fig. 10:**
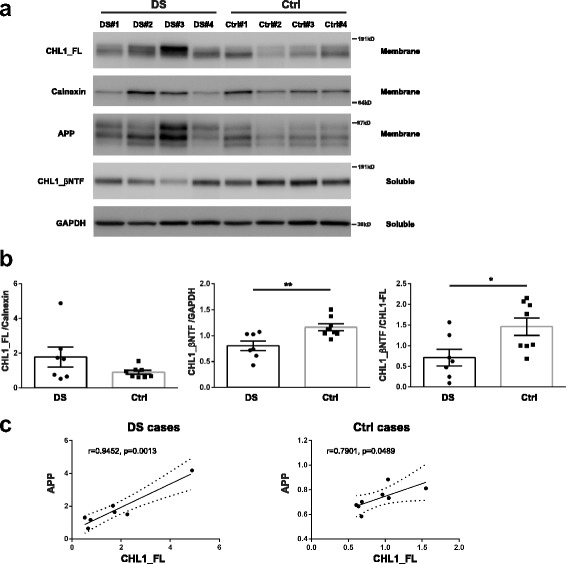
BACE1-mediated cleavage of CHL1 is reduced in human brains from subjects affected by Down syndrome. **a** Representative immunoblot of TBS soluble fraction (Soluble) and membrane fraction (Membrane) of the human hippocampus samples from subjects with Down Syndrome (DS) or unaffected Controls (Ctrl) using anti-human CHL1 (MAB2126), anti-calnexin (610523), and anti-GAPDH (MAB374) antibodies. **b** Graphs represent the ratio CHL1_FL/Calnexin, CHL1_βNTF/GAPDH, and CHL1βNTF/CHL1_FL in five different genotypes. All graphs represent mean ± SEM. Unpaired t-test with Welch’s correction was performed. * p < 0.05, ** p < 0.01 **c** Linear correlation analysis between CHL1_FL and APP levels in Down Syndrome and Control cases. CHL1_FL levels are significantly correlated with membrane-bound APP levels in both DS and Ctrl cases. The dotted line represents 95% confidence interval

In order to further support our finding that increased levels of APP are associated with decreased BACE1-mediated processing of CHL1, we analyzed hippocampal tissue from subjects affected by Down Syndrome (DS) carrying an extra copy of chromosome 21. The APP gene lies on chromosome 21 and as a consequence, levels of APP are increased in the brain of DS patients compared to controls. Soluble and membrane fractions were prepared from snap frozen hippocampus samples. Western blot analysis revealed a decrease in CHL1_βNTF/CHL1_FL ratio in brains from DS patients compared to controls owing to a significant decrease in CHL1_βNTF (Fig. 10a and b). Levels of CHL1_FL were increased but not reached a significant difference in DS vs. control samples. However, a direct correlation was observed between APP and CHL1_FL both in DS and control samples (Fig. 10c). Altogether these results indicate that increased APP levels reduce CHL1 processing not only in the brain of 5XFAD mice but also in human DS brains.

## Discussion

**Fig. 11:**
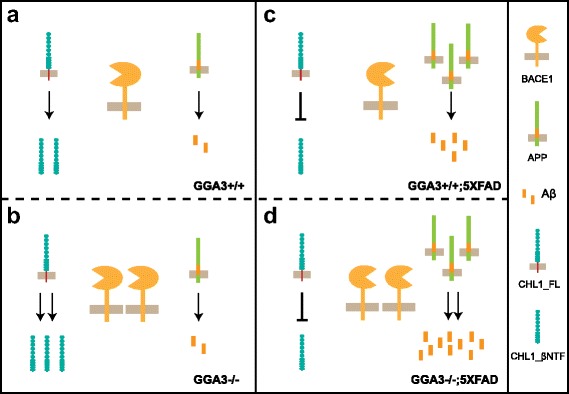
Schematic diagram of BACE1-mediated processing of CHL1 and APP in old mice. **a** BACE1 cleaves both APP and CHL1, resulting in the production of Aβ and N-terminal fragment of CHL1 (CHL1_βNTF), respectively, in GGA3WT mice. **b** Levels of BACE1 are increased in GGA3KO mice, resulting in enhanced processing of CHL1 but not APP. **c** Levels of transgenic APP increase significantly in GGA3WT;5XFAD mice, resulting in elevated production of Aβ. Conversely, CHL1_βNTF/CHL1_FL ratio is decreased because CHL1_FL is outcompeted by APP for BACE1 cleavage and thus stabilized in GGA3WT;5XFAD mice. **d** In GGA3KO;5XFAD mice, increased levels of transgenic APP are associated with elevation of BACE1 due to GGA3 deletion, furthering the accumulation of Aβ and the suppression of CHL1_FL cleavage observed in GGA3WT;5XFAD mice

In this study, we assessed the effect of GGA3 deletion on AD-like phenotypes in 5XFAD mice and found that hippocampal levels of GGA3 decreased while BACE1 levels increased with age in 5XFAD mice, similar to what is observed in human AD brains. Furthermore, GGA3 deletion prevented age-dependent elevation of BACE1 in 5XFAD mice. We also found that GGA3 deletion resulted in increased hippocampal levels of Aβ42 and amyloid burden in 5XFAD mice at 12 months of age. While levels of BACE1 did not change with age in GGA3KO;5XFAD, APP levels increased with age, and were higher in female than male 5XFAD mice. Accordingly, the increase in the hippocampal amyloid burden was more pronounced in female compared to male GGA3KO;5XFAD mice. Moreover, the age- and gender-dependent elevation of APP was associated with a decreased BACE1-mediated processing of endogenous CHL1, most likely owing to substrate competition (Fig. 11).

BACE1 protein levels and β-secretase activity are increased in human AD brains [13, 29–34] and in mouse models of AD [13, 24, 35, 36]. To date, several transcriptional and post-transcriptional mechanisms have been reported to regulate BACE1 levels [37–39]. Some of these mechanisms have been proposed to explain the increased accumulation of BACE1 observed in AD brains, including depletion of GGA3 [8], increased phosphorylation of translation factor eIF2α [40], increased expression of a non-coding anti-sense BACE1 transcript [41] and decreased expression of the BACE1 regulating microRNA’s, miR-29 and miR-107 [42, 43]. Additionally, increasing evidence suggests that BACE1 is a stress-induced protease [39, 44]. BACE1 levels have been shown to increase in cells exposed to oxidative stress [45–48], apoptosis [8], in in vivo animal models following TBI [10, 49, 50], cerebral ischemia [8, 51] and impaired energy metabolism [52]. As changes in BACE1 mRNA levels did not accompany BACE1 protein increases in AD brains in the majority of the studies, post-translational mechanisms are most likely responsible for BACE1 elevation in AD. Furthermore, BACE1-YFP expressed from a transgene lacking the BACE1 mRNA 5’ UTR was also elevated and accumulated around amyloid plaques in 5XFAD brain, suggesting that BACE1 elevation also occurs via a post-translational mechanism in 5XFAD mice [53].

We have previously shown that BACE1 increases following caspase activation both in cellular models of apoptosis and in rodent models of stroke and TBI and proposed that caspase-mediated depletion of GGA3 is the underlying mechanism of BACE1 elevation [8, 10]. More importantly, we discovered that levels of GGA3 were decreased and inversely correlated with BACE1 levels in post-mortem AD brains concurrently with caspase-3 activation [8].

While caspase activation is a well-known mechanism of programmed cell death following TBI in both humans and experimental models [54–57], the role of caspase activation in neurodegenerative diseases has been a matter of debate for a very long time. However, increasing evidence is accumulating of a role for caspase activation not only in AD but also in age-dependent memory impairment [58–60]. Moreover, recent reports have provided compelling evidence that caspase activation is an early event, which plays a key role in neurodegeneration [61, 62]. Eimer and Vassar [63] reported that caspase-3 activation is detected in pyramidal neurons accumulating intraneuronal Aβ42 in the layer 5 of cortex and subiculum in young (1.5 and 4 months of age) 5XFAD mice. Given that neuronal loss appears in the same areas of the brain in old 5XFAD mice, the authors suggested that intraneuronal Aβ42 triggers caspase-3 activation, which in turn induces apoptosis-mediated neurodegeneration and eventual neuronal loss in 5XFAD mice [63]. Accordingly, we found that GGA3 levels are depleted in 5XFAD mice with age while BACE1 levels increase. In further support of the role of GGA3 in the elevation of BACE1 in AD, we found that GGA3 deletion prevented the age-dependent elevation of BACE1 in 5XFAD mice ruling out a major role for other mechanisms implicated in BACE1 regulation. Altogether our data suggest that caspase-mediated depletion of GGA3 is a leading candidate mechanism underlying BACE1 elevation in AD.

In spite of the similar levels of BACE1 in the GGA3KO;5XFAD mice at all ages analyzed (2, 4, 7, 12 months), an increase in Aβ42 levels and amyloid plaques was only observed at 12 months of age. Such increase is most likely due to a significant elevation of full-length APP observed with age in 5XFAD mice [64]. A similar age-dependent increase in APP levels was also observed in APPswe/PS1∆E9, ruling out the possibility that this is an exclusive phenotype of the 5XFAD mice. We also found that both APP and Aβ42 accumulation is more pronounced in female GGAKO;5XFAD mice than in males at 12 months of age. Our findings are in agreement with a previous study showing that female 5XFAD mice have higher steady-state transgenic APP levels than males and proposing that such elevation is due to the presence of an ERE in the 5′ upstream regulatory region of the murine Thy-1 transgene promoter [15]. However, a recent study has reported that levels of full-length APP are higher in females than males also in APP transgenic mice driven by PrP promoter, suggesting that sex-related differences in levels of APP are promoter-independent [65]. While BACE1 seems to be in excess over transgenic APP in young mice (4 months of age) failing to increase Aβ deposition, the processing of another BACE1 substrate, CHL1, was increased in GGA3KO;5XFAD mice. In contrast, we found that CHL1 processing was decreased in 12 months old 5XFAD mice concurrently with a significant increase in APP levels, which was more pronounced in females. Mounting evidence shows that CHL1 is a major player not only in the nervous system development but also in the adult brain during neuronal repair and synaptic plasticity [66, 67]. Increased neuronal expression levels of CHL1 favors axon regeneration after spinal cord, brain and peripheral nerve injury [67]. CHL1 is also found in subpopulations of astrocytes, oligodendrocyte precursors, and Schwann cells [68]. CHL1 expression is increased in reactive astrocytes found at the glial scar, though it inhibits functional recovery after spinal cord injury [69]. Thus, it is possible that the inflammation and gliosis in old 5XFAD mice results in increased expression of CHL1. However, the levels of Chl1 mRNA were similar in the hippocampus of 5XFAD mice compared with non-5XFAD mice ruling out an increase in the expression of CHL1 in aged 5XFAD mice. In addition, levels of full-length CHL1 were similar in GGA3WT;5XFAD and GGA3KO;5XFAD mice while BACE1 levels were increased only in GGA3KO;5XFAD mice. Such BACE1 increase is similar at all age analyzed but results in increased amyloid burden only at 12 months of age when levels of APP are significantly elevated. Thus, another possible explanation of BACE1-mediated processing of CHL1 is that high levels of transgenic APP compete with CHL1 for BACE1 processing.

BACE1 elevation engendered by GGA3 deletion was sufficient to increase CHL1 processing at both 4 and 12 months of age in GGA3KO mice. However, we have previously reported that endogenous Aβ levels do not increase in GGA3KO mice [10]. Altogether, these data suggest that BACE1 preferentially cleaves CHL1 over endogenous APP. Given that levels of BACE1 in GGA3KO mice were similar to GGA3KO;5XFAD mice, it appears that neither endogenous APP nor transgenic APP levels were sufficient enough to outcompete other substrate for BACE1-mediated processing at a young age (Fig. 11). Our findings are in agreement with previous work showing that CHL1 is primarily processed by BACE1 while APP is primarily processed by ADAMs (a disintegrin and metalloproteases) [26]. In further support of our finding that increased levels of APP are associated with decreased BACE1-mediated processing of CHL1, we have found that CHL1_βNTF/CHL1_FL ratio is decreased in DS brains.

Increased levels of APP are associated with AD not only in human subjects with Trisomy 21 [70] but also with APP duplication (Finnish mutation) [71]. Alzheimer’s disease is more common in women. Similarly, a significant age and sex dependent difference of Aβ immunoreactivity has been observed in several mouse model of AD, including 5XFAD, APPswe/PS1ΔE9 mice and more recently in 18 months old APP^NL-G-F/NL-G-F^ knock-in mice [72]. A possible explanation for increased levels of APP in old and female 5XFAD mice is Aβ toxicity. Elevation of APP has been reported to be induced by Aβ toxicity both in vitro and in vivo [53, 73]. Accordingly, APP accumulation has been observed in plaques-associated dystrophic neurites in AD brains [74, 75] and 5XFAD mice [13, 35, 76].

Similarly, BACE1 accumulates in swollen dystrophic neurites in very close proximity to amyloid plaques in the brain of APP transgenic mice and AD patients [13, 24, 35, 36], suggesting that Aβ toxicity results in BACE1 elevation. In support of this hypothesis, Aβ treatment induces BACE1 elevation in neuronal cultures [53, 77–79]. Thus, it is likely that the progressive accumulation of Aβ during aging induces APP and BACE1 elevation establishing a vicious cycle in 5XFAD mice. Moreover, in spite of the elevation of BACE1 it is possible that some of the phenotypes of the aged 5XFAD mice could be due to a decreased processing of other BACE1 substrates at synaptic sites owing to APP competition for BACE1 cleavage.

BACE1 is a primary drug target for AD therapy. Phase II/III trials are currently ongoing for selective active site inhibitors of BACE [80]. While these drugs show initial promise for the treatment of AD, it remains unclear whether they will remain viable in future trials owing to mechanism-based side effects [81]. Given that BACE1 cleaves preferentially other substrates over APP, the degree of BACE1 inhibition required to reduce Aβ levels may be sufficient to severely impair the processing of other BACE1 substrates. More importantly, APP elevation could further potentiate the effect of BACE inhibition on the processing of substrates like CHL1 and increase the possibility of mechanism-based site effects.

Thus, the identification of the molecular mechanism (s) (e.g. GGA3 depletion) responsible for BACE1 elevation may lead to the identification of BACE1 lowering strategies that can represent an attractive alternative to BACE inhibition for the prevention/treatment of AD. A possible therapeutic approach may be represented by the preservation and/or increase of GGA3 levels. Small molecules called pharmacological chaperones are able to increase protein levels by preventing their degradation [82]. Pharmacological chaperones able to stabilize the retromer complex and limit APP processing have been recently identified [82]. This approach has the advantage to restore the function of the affected protein (e.g. GGA3) and to prevent the down stream effects of its depletion (e.g. BACE1 elevation). Furthermore, a better understanding of GGA3-mediated degradation of BACE1 may lead to identification of small molecules in the future. For example, we have demonstrated that GGA3 regulates BACE1 degradation via interaction with ubiquitin and that BACE1 is ubiquitinated [9, 83]. More recently, we have identified the endosomal-associated deubiquitinating enzyme USP8 as a negative regulator of BACE1 ubiquitination and degradation [84]. Thus, although USP8 serves some essential functions, in disorders associated with accumulation of BACE1, it may be a potential therapeutic target for small-molecule inhibitors. Interestingly, several small molecule inhibitors for specific DUBs and screens to find such inhibitors have recently been developed [85–87].

## Conclusions

In this study, we assessed the effect of GGA3 deletion on AD-like phenotypes in 5XFAD mice and found that hippocampal levels of GGA3 decreased while BACE1 levels increased with age in 5XFAD mice, similar to what is observed in human AD brains. Furthermore, GGA3 deletion prevented age-dependent elevation of BACE1 in 5XFAD mice. We also found that GGA3 deletion resulted in increased hippocampal levels of Aβ42 and amyloid burden in 5XFAD mice at 12 months of age. While levels of BACE1 did not change with age in GGA3KO;5XFAD, APP levels increased with age, and were higher in female than male 5XFAD mice. Accordingly, the increase in the hippocampal amyloid burden was more pronounced in female compared to male GGA3KO;5XFAD mice. Moreover, the age- and gender-dependent elevation of APP was associated with a decreased BACE1-mediated processing of endogenous CHL1, most likely owing to substrate competition. In further support of these findings, we found that elevation of APP was associated with a decreased BACE1-mediated processing of CHL1 in human brains from subjects affected by Down syndrome.

## Additional files

Additional file 1: Comparison of Aβ42 levels and amyloid burden in male and female 5XFAD mice at 4 months of age. The graphs represent levels of human Aβ42 (A) and percentage of area occupied by Thioflavin-S positive plaques (B) in hippocampus and cortex from male and female GGA3WT;5XFAD, GGA3Het;5XFAD and GGA3KO;5XFAD mice at 4 months of age. Levels of Aβ42 (A) and amyloid burden (B) were significantly higher in females than in males with the same genotype. Total number of mice in each group is indicated within bars. All graphs represent mean ± SEM. Two-way ANOVA with Fisher’s LSD post hoc tests was applied to each sex group. #### < 0.0001. (PDF 666 kb)

Additional file 2: GGA3 deletion does not increase levels of BACE1-generated APP-CTFs at 4 months of age in 5XFAD mice.(A) Representative immunoblots of hippocampus (left) and cortex (right) homogenates from 4 months old GGA3WT;5XFAD, GGA3Het;5XFAD, and GGA3KO;5XFAD mice probed with anti-APP C-terminal (C1/6.1) and anti-GAPDH (MAB374) antibodies. C99 and C89 fragments are BACE1-mediated APP C-terminal fragments (APP-CTFs), while C83 fragment is alpha-secretase-mediated APP-CTF. APP-CTFs are present as phosphorylated (pC99, pC89, and pC83) and nonphosphorylated (C99, C89, and C83) forms. (B) Densitometry levels of full-length APP (fAPP), pC99, C99, and pC89 were quantified, and normalized to GAPDH or fAPP. Table shows the summary of total APP levels (fAPP/GAPDH) and BACE1-mediated processing of APP (pC99/fAPP, C99/fAPP, pC89/fAPP) in hippocampus and cortex homogenates from 4 months old GGA3WT;5XFAD, GGA3Het;5XFAD, and GGA3KO;5XFAD mice. One-way ANOVA with Fisher’s LSD post hoc tests was applied to each genotype group. (PDF 543 kb)

Additional file 3: Comparison of Aβ42 levels and amyloid burden in male and female 5XFAD mice at 12 months of age. The graphs represent human Aβ42 levels (A) and quantification of Thioflavin-S positive plaques (B-D) in hippocampus and cortex from 12 months old GGA3WT;5XFAD, GGA3Het;5XFAD and GGA3KO;5XFAD male and female mice. Levels of Aβ42 are significantly higher in hippocampus and cortex from females than from males with the same genotype (A). Amyloid burden was not significantly different between males and females, except in the cortex from GGA3KO;5XFAD mice (B). (C-D) The graphs represent the average size of amyloid plaques (μm^2^) and the number of amyloid plaques in hippocampus (C) and cortex (D) of GGA3WT;5XFAD and GGA3KO;5XFAD mice. The number of amyloid plaques, but not their size, was significantly higher in the cortex of females compared to males. Total number of mice in each group is indicated within bars. All graphs represent mean ± SEM. Two-way ANOVA with Fisher’s LSD post hoc tests was applied to each sex group. # *p* < 0.05, ## *p* < 0.01, #### < 0.0001. (PDF 1118 kb)

Additional file 4: Levels of transgenic human APP increase with age in APPswe/PS1ΔE9 mice. Representative immunoblot of hippocampus and cortex homogenates from 4 and 10 months old APPswe/PS1ΔE9 female mice probed with anti-APP C-terminal (C1/6.1) and anti-GAPDH (MAB374) antibodies. Increased BACE1 levels are observed in cortex, but not in hippocampus homogenates from 10 months of APPswe/PS1ΔE9 mice compared to 4 months old mice. Old APPswe/PS1ΔE9 mice have significantly increased transgenic APP levels in both hippocampus and cortex compared to 4 months old mice. (PDF 525 kb)

Additional file 5: GGA3 deletion does not increase levels of BACE1-generated APP-CTFs age in 12 months old 5XFAD mice. (A) Representative immunoblots of hippocampus homogenates from 12 months old GGA3WT;5XFAD, GGA3Het;5XFAD, and GGA3KO;5XFAD males (left) and females (right) probed with anti-APP C-terminal (C1/6.1) and anti-GAPDH (MAB374) antibodies. (B) Densitometry levels of full-length APP (fAPP), pC99, C99, and pC89 were quantified, and normalized to GAPDH or fAPP. Table shows the summary of total APP levels (fAPP/GAPDH) and BACE1-mediated processing of APP (pC99/fAPP, C99/fAPP, pC89/fAPP) in hippocampus homogenates from 12 months old GGA3WT;5XFAD, GGA3Het;5XFAD, and GGA3KO;5XFAD mice. One-way ANOVA with Fisher’s LSD post hoc tests was applied to each genotype group. (PDF 531 kb)

Additional file 6: Detection of soluble and membrane-bound CHL1 fragments in mouse hippocampus. Representative immunoblot of soluble and membrane fractions compared to total protein extracts from BACE1WY, BACE1Het and BACE1KO mice. Snap frozen hippocampi from 12 months old BACE1WT, BACE1Het, and BACE1KO mice were separated in PBS soluble fraction (Soluble), membrane fraction (Membrane), and total protein extract (Total) as described in method. Samples were separated in 3–8% Tris-acetate gels to detect CHL1_FL and CHL1_NTF using anti-CHL1 (AF2147) antibody. Western blot analysis of CHL1 clearly detected two membrane bound CHL1 fragments in the membrane fraction, corresponding to CHL1_FL (~ 185 kDa) and the ~ 175 kDa band (arrowhead) also detected in the total extract (Figs. 4b and 9a). CHL1_FL levels were increased in BACE1KO in the membrane fraction and total extract. CHL1_βNTF was detected in the soluble fraction and corresponds to the ~ 165 kDa band detected in the total extract. A fragment of lower molecular weight (indicated by an asterisk) was detected in the soluble fraction from BACE1KO mice. Such soluble fragment is most likely derived by a compensatory increased cleavage of CHL1 by ADAM8 or ADAM10. As expected membrane proteins including calnexin and BACE1 were absent in the soluble fraction. (PDF 1089 kb)

Additional file 7: BACE1-mediated cleavage of CHL1 is reduced in old 5XFAD mice. (A) Representative immunoblot of PBS soluble fraction (Soluble) and membrane fraction (Membrane) from the hippocampus of BACE1KO, BACEHet, wild type (indicated as GGA3WT), GGA3KO, 5XFAD, and BACE1Het;5XFAD mice using anti-CHL1 (AF2147), anti-calnexin (610523), anti-BACE1 (D10E5), and anti-GAPDH (MAB374) antibodies. Increased levels of CHL1_FL were observed in mice with reduced BACE1 (BACE1Het, BACE1Het;5XFAD, and BACE1KO) compared to GGA3WT mice while a decrease in CHL1_FL levels was only observed in mice with elevated BACE1 (GGA3KO). Levels of CHL1_FL were increased in old 5XFAD mice compared with wild type mice. Soluble CHL1_βNTF was also increased in 5XFAD mice compared to wild type mice. A soluble CHL1 fragment was detected in BACEKO mice (asterisk), which may be derived by compensatory increased ADAM8 or ADAM 10 cleavage. (B) Graphs represent the ratio CHL1_FL/Calnexin, CHL1_βNTF/GAPDH, and CHL1_βNTF/CHL1_FL in five different genotypes. GGA3KO mice showed significantly increased ratio of CHL1_βNTF/CHL1_FL compared with GGA3WT mice. BACE1Het, 5XFAD, and BACE1Het;5XFAD mice exhibited reduced ratio of CHL1_βNTF/CHL1_FL, suggesting these mice has reduced BACE1-mediated CHL1 processing. Total number of mice in each group is indicated within bars. All graphs represent mean ± SEM. One-way ANOVA with Fisher’s LSD post hoc tests were applied to each genotype group. * *p* < 0.05, *** *p* < 0.001, **** < 0.0001. (PDF 1248 kb)

Additional file 8: CHL1 expression is similar in 5XFAD and 5XFAD mice. (A) mRNA expression levels for CHL1 were analyzed in the hippocampus of non-5XFAD (non-5XFAD) and 5XFAD (5XFAD) mice by RT-qPCR. 5XFAD mice had similar level of mRNA Chl1. (B) mRNA Chl1 levels were analyzed in the hippocampus of 5XFAD males and females, indicating no sex difference in 5XFAD mice. Total number of mice in each group is indicated within bars. All graphs represent mean ± SEM. Unpaired t-test with Welch’s correction was performed. (PDF 337 kb)

## Abbreviations

GGA3: Golgi-localized γ-ear-containing ARF binding protein 3
AD: Alzheimer’s disease
ADAMs: A disintegrin and metalloproteases
APP: Amyloid precursor protein
Aβ: Amyloid beta
BACE1: β-site amyloid precursor protein cleaving enzyme 1
CA1so: CA1 stratum oriens
CHL1: Cell adhesion molecule L1 like protein
CHL1_FL: Full-length CHL1
CHL1_βNTF: N-terminal CHL1 fragment
DG: Dentate gyrus
FAD: Familial Alzheimer’s Disease
GuHCl: Guanidine hydrochloride
PS1: Presenilin 1
RIPA: Radioimmunoprecipitation assay
slu: Stratum lucidum
TBI: Traumatic brain injury
TGN: Trans-Golgi network
