# Supplementary figures and images for: BACE1 elevation engendered by GGA3 deletion increases β-amyloid pathology in association with APP elevation and decreased CHL1 processing in 5XFAD mice
Source: Mol Neurodegener. 2018 Feb 2;13:6. doi: 10.1186/s13024-018-0239-7 (PMC5796504; doi:10.1186/s13024-018-0239-7)

**A**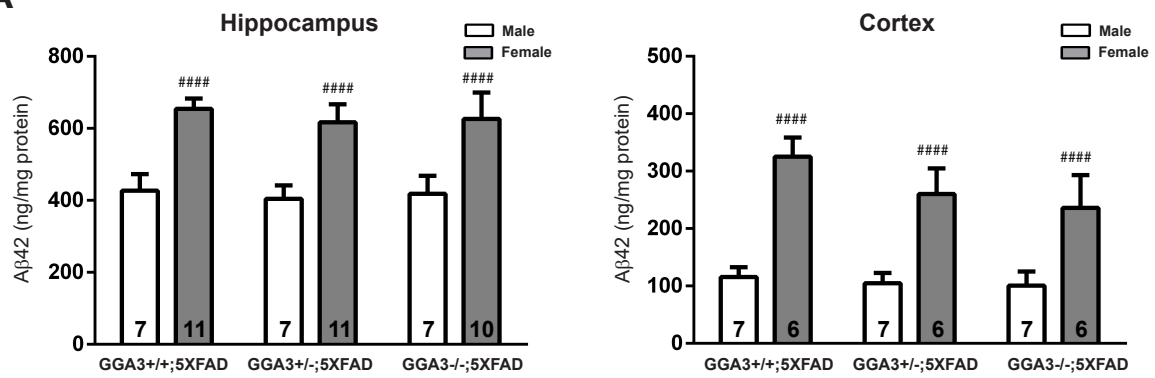**B**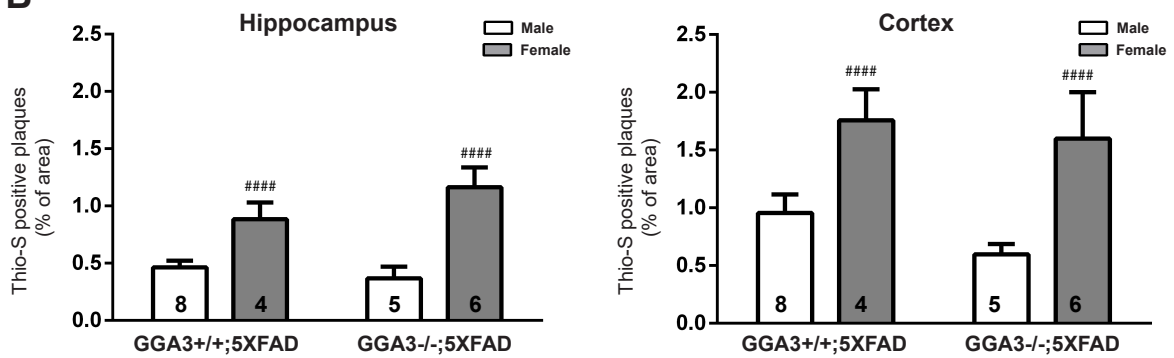**Additional File 1**

Supplement: Supplementary file 1 — Comparison of Aβ42 levels and amyloid burden in male and female 5XFAD mice at 4 months of age. The graphs represent levels of human Aβ42 (A) and percentage of area occupied by Thioflavin-S positive plaques (B) in hippocampus and cortex from male and female GGA3WT;5XFAD, GGA3Het;5XFAD and GGA3KO;5XFAD mice at 4 months of age. Levels of Aβ42 (A) and amyloid burden (B) were significantly higher in females than in males with the same genotype. Total number of mice in each group is indicated within bars. All graphs represent mean ± SEM. Two-way ANOVA with Fisher’s LSD post hoc tests was applied to each sex group. #### < 0.0001. (PDF 666 kb) [file 13024_2018_239_MOESM1_ESM.pdf]

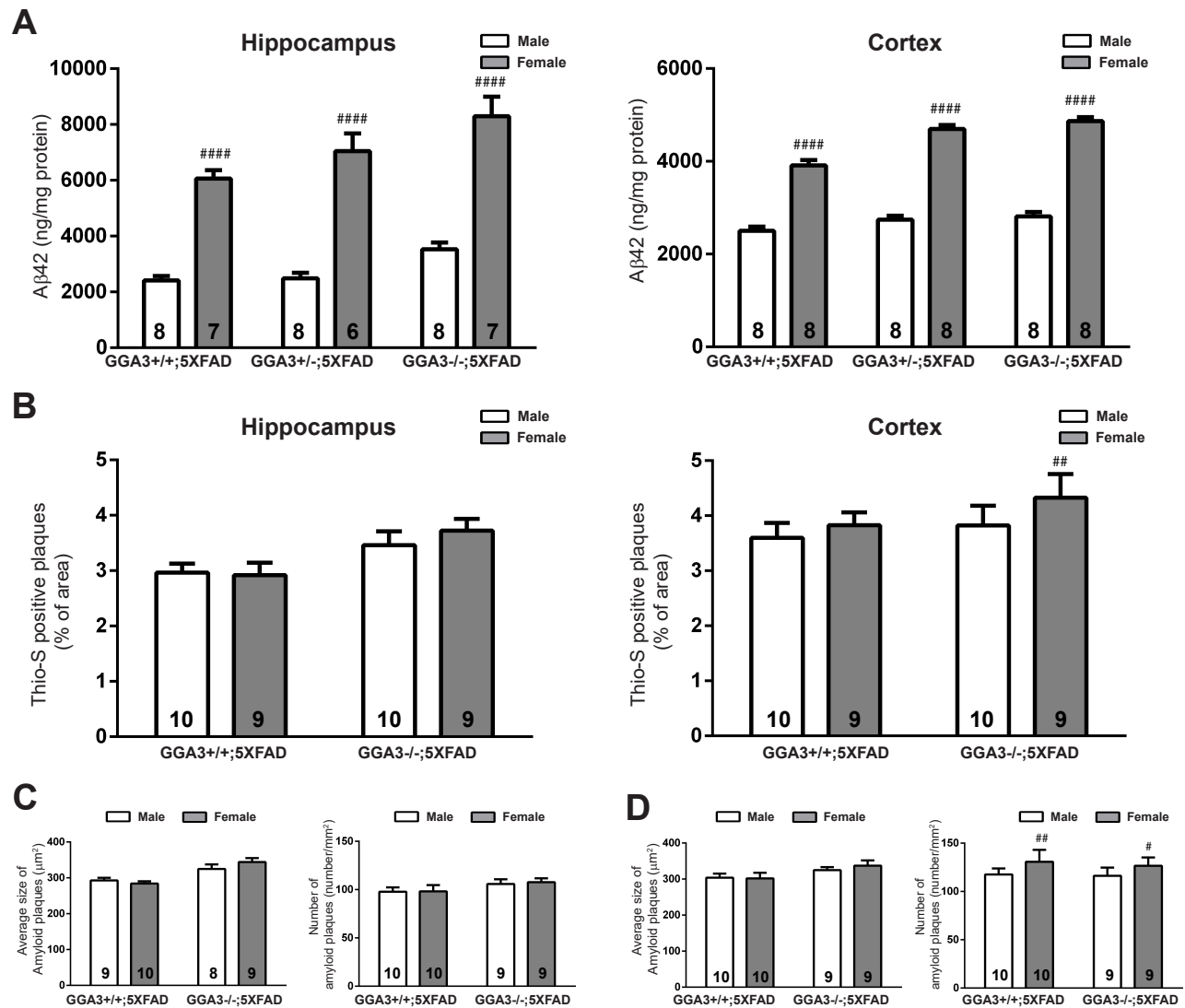

**Additional File 3**

Supplement: Supplementary file 3 — Comparison of Aβ42 levels and amyloid burden in male and female 5XFAD mice at 12 months of age. The graphs represent human Aβ42 levels (A) and quantification of Thioflavin-S positive plaques (B-D) in hippocampus and cortex from 12 months old GGA3WT;5XFAD, GGA3Het;5XFAD and GGA3KO;5XFAD male and female mice. Levels of Aβ42 are significantly higher in hippocampus and cortex from females than from males with the same genotype (A). Amyloid burden was not significantly different between males and females, except in the cortex from GGA3KO;5XFAD mice (B). (C-D) The graphs represent the average size of amyloid plaques (μm2) and the number of amyloid plaques in hippocampus (C) and cortex (D) of GGA3WT;5XFAD and GGA3KO;5XFAD mice. The number of amyloid plaques, but not their size, was significantly higher in the cortex of females compared to males. Total number of mice in each group is indicated within bars. All graphs represent mean ± SEM. Two-way ANOVA with Fisher’s LSD post hoc tests was applied to each sex group. # p < 0.05, ## p < 0.01, #### < 0.0001. (PDF 1118 kb) [file 13024_2018_239_MOESM3_ESM.pdf]

**APP<sup>swe</sup>/PS1<sup>ΔE9</sup>**

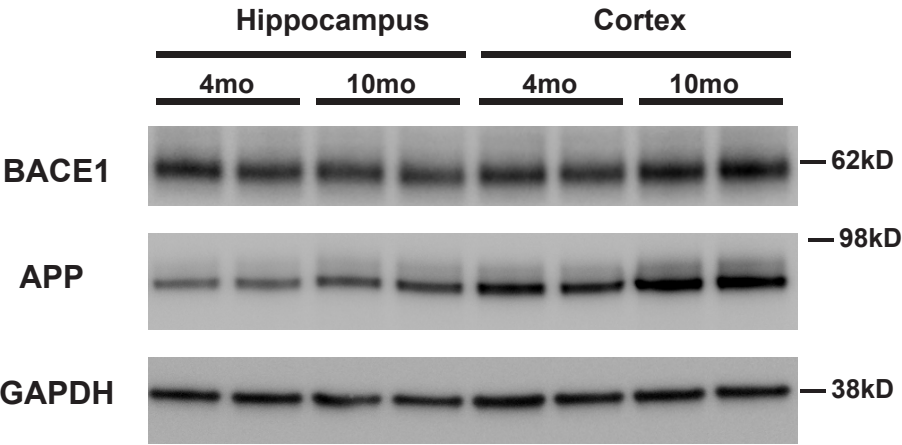

**Additional File 4**

Supplement: Supplementary file 4 — Levels of transgenic human APP increase with age in APPswe/PS1ΔE9 mice. Representative immunoblot of hippocampus and cortex homogenates from 4 and 10 months old APPswe/PS1ΔE9 female mice probed with anti-APP C-terminal (C1/6.1) and anti-GAPDH (MAB374) antibodies. Increased BACE1 levels are observed in cortex, but not in hippocampus homogenates from 10 months of APPswe/PS1ΔE9 mice compared to 4 months old mice. Old APPswe/PS1ΔE9 mice have significantly increased transgenic APP levels in both hippocampus and cortex compared to 4 months old mice. (PDF 525 kb) [file 13024_2018_239_MOESM4_ESM.pdf]

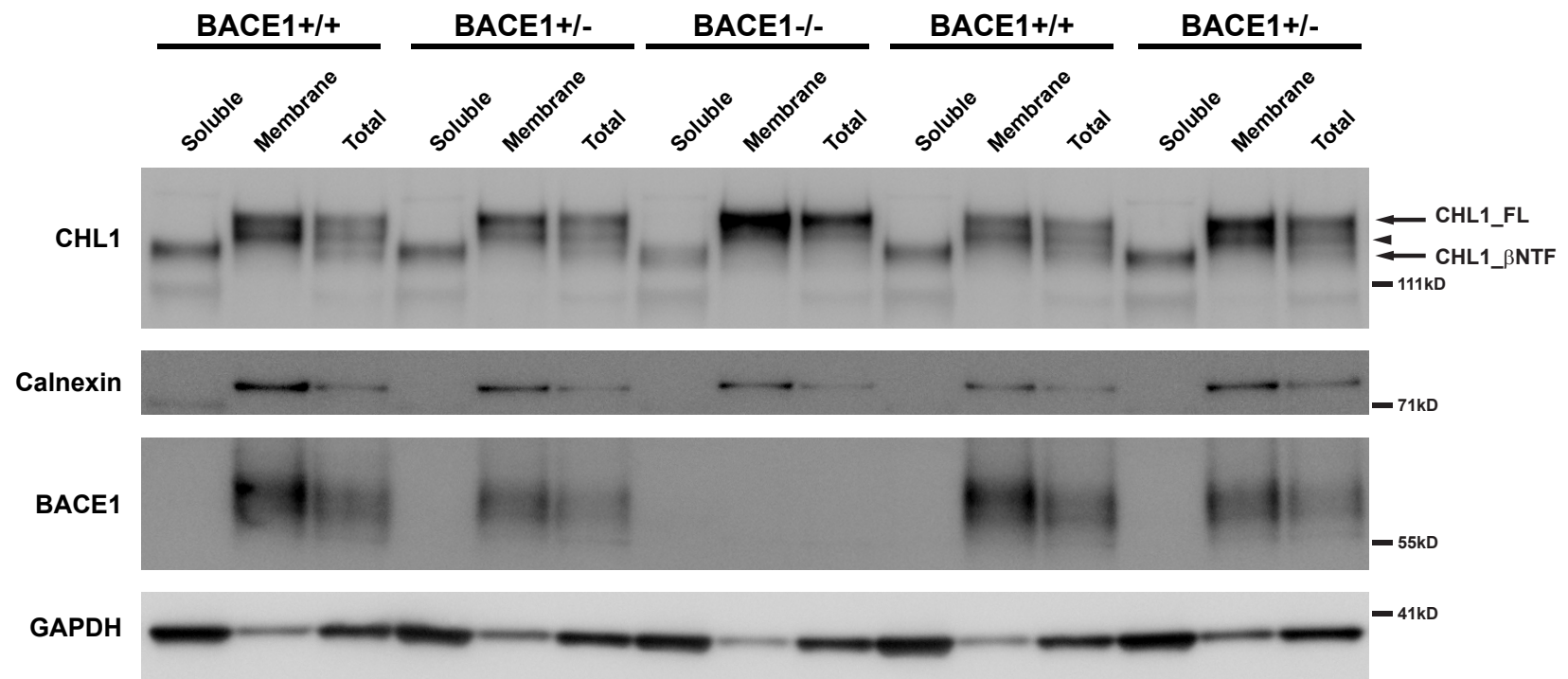

Additional File 6

Supplement: Supplementary file 6 — Detection of soluble and membrane-bound CHL1 fragments in mouse hippocampus. Representative immunoblot of soluble and membrane fractions compared to total protein extracts from BACE1WY, BACE1Het and BACE1KO mice. Snap frozen hippocampi from 12 months old BACE1WT, BACE1Het, and BACE1KO mice were separated in PBS soluble fraction (Soluble), membrane fraction (Membrane), and total protein extract (Total) as described in method. Samples were separated in 3–8% Tris-acetate gels to detect CHL1_FL and CHL1_NTF using anti-CHL1 (AF2147) antibody. Western blot analysis of CHL1 clearly detected two membrane bound CHL1 fragments in the membrane fraction, corresponding to CHL1_FL (~ 185 kDa) and the ~ 175 kDa band (arrowhead) also detected in the total extract (Figs. 4b and 9a). CHL1_FL levels were increased in BACE1KO in the membrane fraction and total extract. CHL1_βNTF was detected in the soluble fraction and corresponds to the ~ 165 kDa band detected in the total extract. A fragment of lower molecular weight (indicated by an asterisk) was detected in the soluble fraction from BACE1KO mice. Such soluble fragment is most likely derived by a compensatory increased cleavage of CHL1 by ADAM8 or ADAM10. As expected membrane proteins including calnexin and BACE1 were absent in the soluble fraction. (PDF 1089 kb) [file 13024_2018_239_MOESM6_ESM.pdf]

**A**

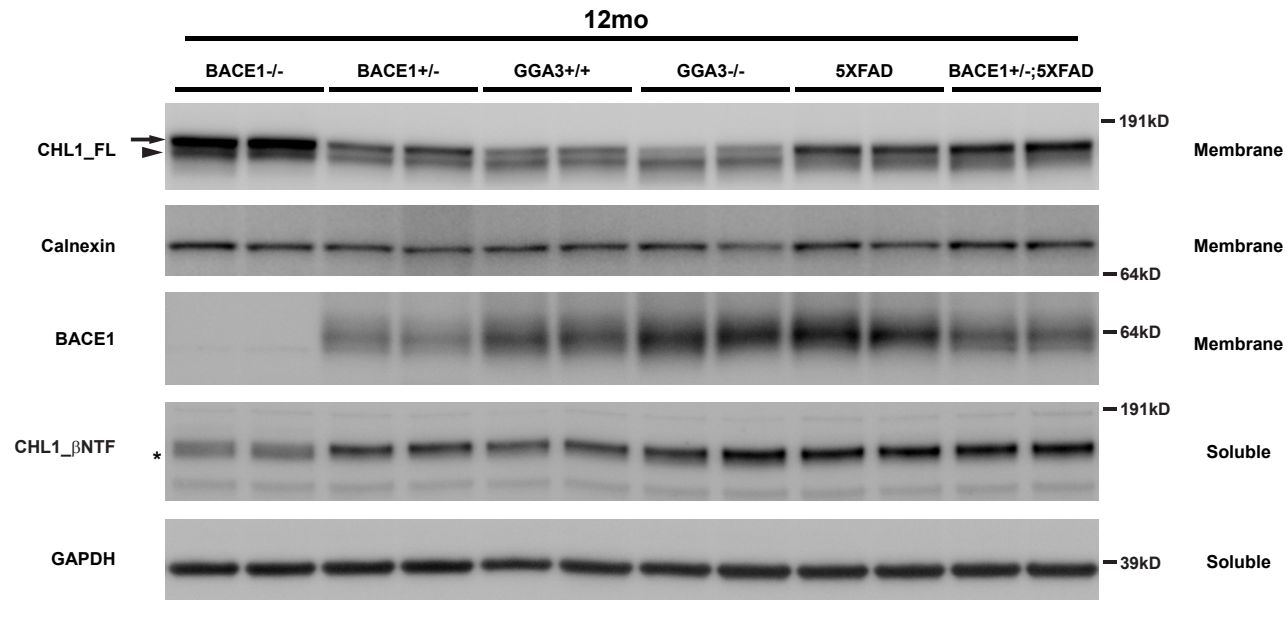

**B**

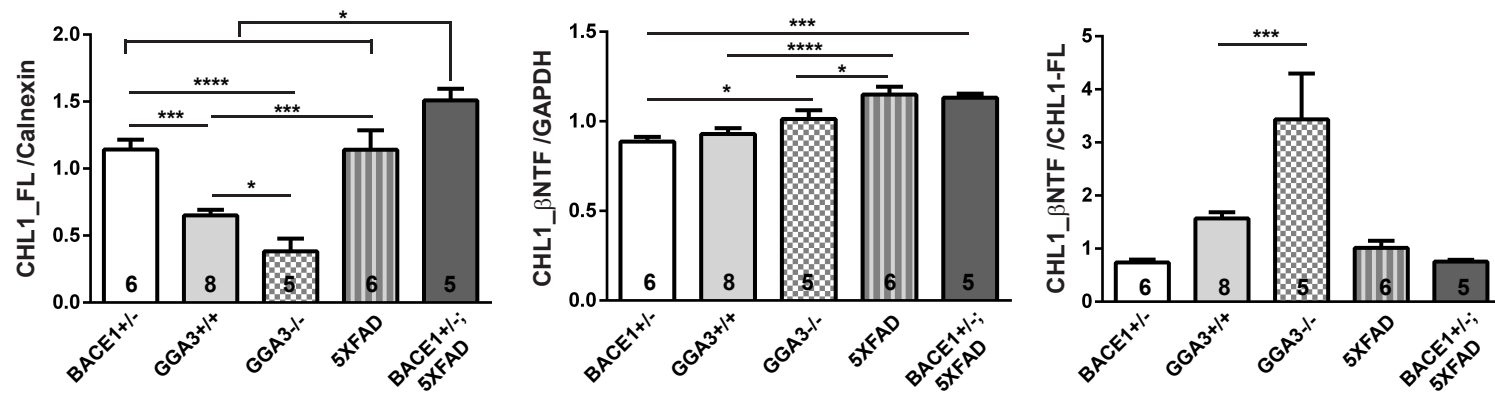

Supplement: Supplementary file 7 — BACE1-mediated cleavage of CHL1 is reduced in old 5XFAD mice. (A) Representative immunoblot of PBS soluble fraction (Soluble) and membrane fraction (Membrane) from the hippocampus of BACE1KO, BACEHet, wild type (indicated as GGA3WT), GGA3KO, 5XFAD, and BACE1Het;5XFAD mice using anti-CHL1 (AF2147), anti-calnexin (610523), anti-BACE1 (D10E5), and anti-GAPDH (MAB374) antibodies. Increased levels of CHL1_FL were observed in mice with reduced BACE1 (BACE1Het, BACE1Het;5XFAD, and BACE1KO) compared to GGA3WT mice while a decrease in CHL1_FL levels was only observed in mice with elevated BACE1 (GGA3KO). Levels of CHL1_FL were increased in old 5XFAD mice compared with wild type mice. Soluble CHL1_βNTF was also increased in 5XFAD mice compared to wild type mice. A soluble CHL1 fragment was detected in BACEKO mice (asterisk), which may be derived by compensatory increased ADAM8 or ADAM 10 cleavage. (B) Graphs represent the ratio CHL1_FL/Calnexin, CHL1_βNTF/GAPDH, and CHL1_βNTF/CHL1_FL in five different genotypes. GGA3KO mice showed significantly increased ratio of CHL1_βNTF/CHL1_FL compared with GGA3WT mice. BACE1Het, 5XFAD, and BACE1Het;5XFAD mice exhibited reduced ratio of CHL1_βNTF/CHL1_FL, suggesting these mice has reduced BACE1-mediated CHL1 processing. Total number of mice in each group is indicated within bars. All graphs represent mean ± SEM. One-way ANOVA with Fisher’s LSD post hoc tests were applied to each genotype group. * p < 0.05, *** p < 0.001, **** < 0.0001. (PDF 1248 kb) [file 13024_2018_239_MOESM7_ESM.pdf]

**A**

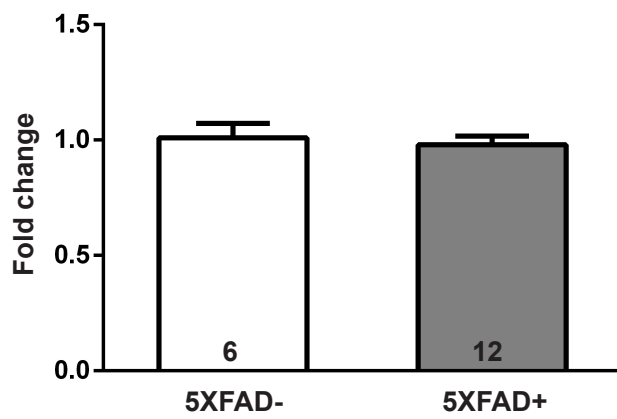

**B**

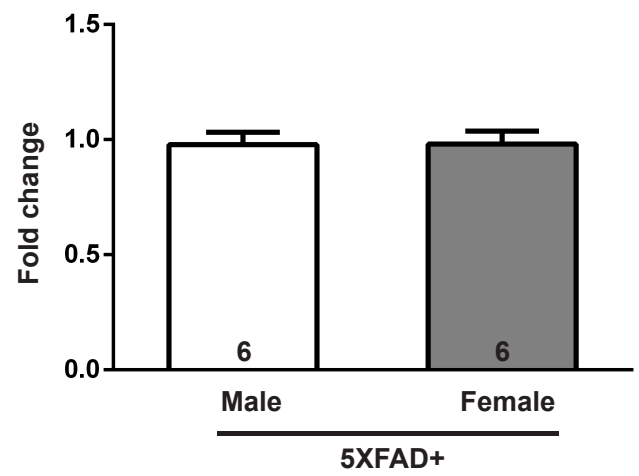

Supplement: Supplementary file 8 — CHL1 expression is similar in 5XFAD and 5XFAD mice. (A) mRNA expression levels for CHL1 were analyzed in the hippocampus of non-5XFAD (non-5XFAD) and 5XFAD (5XFAD) mice by RT-qPCR. 5XFAD mice had similar level of mRNA Chl1. (B) mRNA Chl1 levels were analyzed in the hippocampus of 5XFAD males and females, indicating no sex difference in 5XFAD mice. Total number of mice in each group is indicated within bars. All graphs represent mean ± SEM. Unpaired t-test with Welch’s correction was performed. (PDF 337 kb) [file 13024_2018_239_MOESM8_ESM.pdf]
