# Supplementary material for: BACE1 elevation engendered by GGA3 deletion increases β-amyloid pathology in association with APP elevation and decreased CHL1 processing in 5XFAD mice
Source: Mol Neurodegener. 2018 Feb 2;13:6. doi: 10.1186/s13024-018-0239-7 (PMC5796504; doi:10.1186/s13024-018-0239-7)
Supplement: Supplementary file 2 — GGA3 deletion does not increase levels of BACE1-generated APP-CTFs at 4 months of age in 5XFAD mice.(A) Representative immunoblots of hippocampus (left) and cortex (right) homogenates from 4 months old GGA3WT;5XFAD, GGA3Het;5XFAD, and GGA3KO;5XFAD mice probed with anti-APP C-terminal (C1/6.1) and anti-GAPDH (MAB374) antibodies. C99 and C89 fragments are BACE1-mediated APP C-terminal fragments (APP-CTFs), while C83 fragment is alpha-secretase-mediated APP-CTF. APP-CTFs are present as phosphorylated (pC99, pC89, and pC83) and nonphosphorylated (C99, C89, and C83) forms. (B) Densitometry levels of full-length APP (fAPP), pC99, C99, and pC89 were quantified, and normalized to GAPDH or fAPP. Table shows the summary of total APP levels (fAPP/GAPDH) and BACE1-mediated processing of APP (pC99/fAPP, C99/fAPP, pC89/fAPP) in hippocampus and cortex homogenates from 4 months old GGA3WT;5XFAD, GGA3Het;5XFAD, and GGA3KO;5XFAD mice. One-way ANOVA with Fisher’s LSD post hoc tests was applied to each genotype group. (PDF 543 kb) [file 13024_2018_239_MOESM2_ESM.pdf]

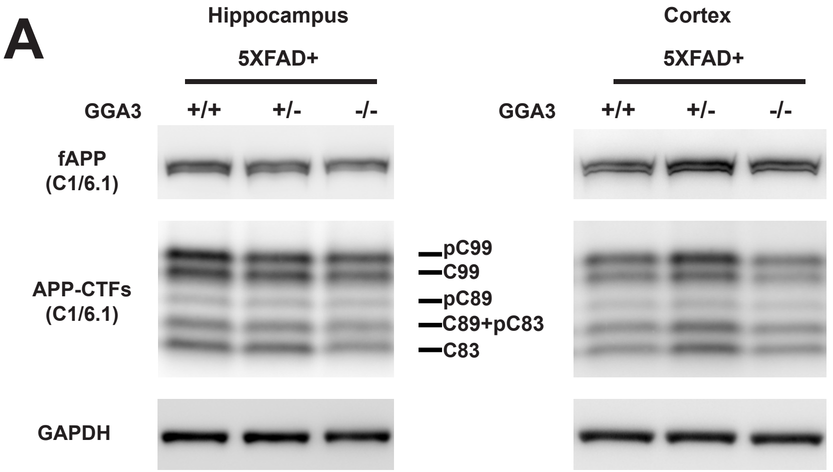

B

Western blot analysis of fAPP and CTFs levels (Mean ± SEM)				
Hippocampus		GGA3+/+;5XFAD (n=8)	GGA3+/-;5XFAD (n=9)	GGA3-/-;5XFAD (n=7)
	fAPP/GAPDH	1.07 ± 0.05	0.94 ± 0.03	0.94 ± 0.04
	pC99/fAPP	1.03 ± 0.05	0.93 ± 0.10	1.05 ± 0.11
	C99/fAPP	1.28 ± 0.13	1.04 ± 0.09	1.05 ± 0.14
	pC89/fAPP	1.00 ± 0.07	0.89 ± 0.13	0.86 ± 0.10
Cortex		GGA3+/+;5XFAD (n=8)	GGA3+/-;5XFAD (n=10)	GGA3-/-;5XFAD (n=8)
	fAPP/GAPDH	1.03 ± 0.07	1.01 ± 0.08	0.99 ± 0.07
	pC99/fAPP	0.95 ± 0.95	0.80 ± 0.09	0.86 ± 0.08
	C99/fAPP	0.94 ± 0.07	0.73 ± 0.07	0.85 ± 0.10
	pC89/fAPP	1.03 ± 0.10	0.84 ± 0.09	0.96 ± 0.11

Additional File 2
