# Supplementary material for: BACE1 elevation engendered by GGA3 deletion increases β-amyloid pathology in association with APP elevation and decreased CHL1 processing in 5XFAD mice
Source: Mol Neurodegener. 2018 Feb 2;13:6. doi: 10.1186/s13024-018-0239-7 (PMC5796504; doi:10.1186/s13024-018-0239-7)
Supplement: Supplementary file 5 — GGA3 deletion does not increase levels of BACE1-generated APP-CTFs age in 12 months old 5XFAD mice. (A) Representative immunoblots of hippocampus homogenates from 12 months old GGA3WT;5XFAD, GGA3Het;5XFAD, and GGA3KO;5XFAD males (left) and females (right) probed with anti-APP C-terminal (C1/6.1) and anti-GAPDH (MAB374) antibodies. (B) Densitometry levels of full-length APP (fAPP), pC99, C99, and pC89 were quantified, and normalized to GAPDH or fAPP. Table shows the summary of total APP levels (fAPP/GAPDH) and BACE1-mediated processing of APP (pC99/fAPP, C99/fAPP, pC89/fAPP) in hippocampus homogenates from 12 months old GGA3WT;5XFAD, GGA3Het;5XFAD, and GGA3KO;5XFAD mice. One-way ANOVA with Fisher’s LSD post hoc tests was applied to each genotype group. (PDF 531 kb) [file 13024_2018_239_MOESM5_ESM.pdf]

A

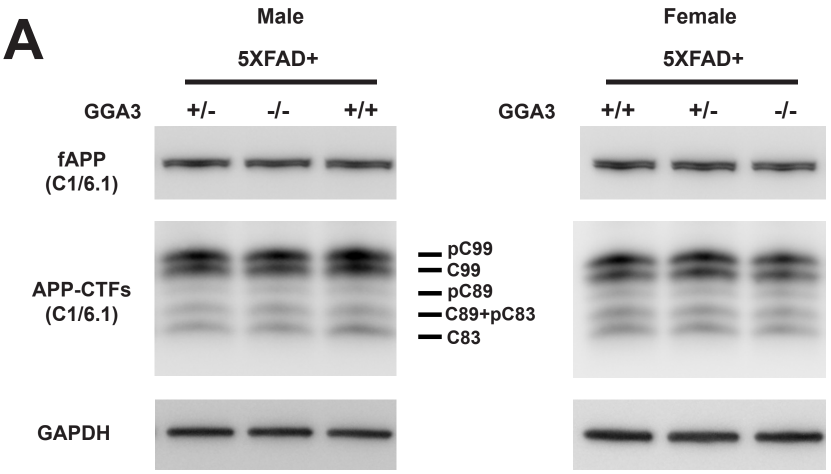

B

Western blot analysis of fAPP and CTFs levels (Mean ± SEM)				
		GGA3+/+;5XFAD (n=8)	GGA3+/-;5XFAD (n=8)	GGA3-/-;5XFAD (n=9)
Male	fAPP/GAPDH	1.09 ± 0.03	0.96 ± 0.03	0.92 ± 0.04
	pC99/fAPP	1.07 ± 0.07	1.05 ± 0.09	1.09 ± 0.06
	C99/fAPP	1.18 ± 0.09	1.03 ± 0.05	1.07 ± 0.05
	pC89/fAPP	1.12 ± 0.05	0.96 ± 0.08	1.14 ± 0.06
		GGA3+/+;5XFAD (n=9)	GGA3+/-;5XFAD (n=8)	GGA3-/-;5XFAD (n=9)
Female	fAPP/GAPDH	1.03 ± 0.04	0.93 ± 0.04	1.05 ± 0.05
	pC99/fAPP	0.96 ± 0.06	1.02 ± 0.05	1.06 ± 0.07
	C99/fAPP	1.00 ± 0.04	1.08 ± 0.04	0.95 ± 0.04
	pC89/fAPP	1.04 ± 0.09	1.01 ± 0.07	1.01 ± 0.09

Additional File 5
